# Active Components of *Ginkgo biloba* Flower Attenuate Radiation-Induced Cognitive Impairment via Inhibiting Ferroptosis

**DOI:** 10.3390/antiox15020183

**Published:** 2026-02-01

**Authors:** Ruihong Li, Yuying Wang, Xin Sun, Ziming Xia, Ying Tian, Biqiong Chen, Shuchen Liu, Min Li, Xinlong Yan

**Affiliations:** 1 Beijing Key Laboratory of Environmental and Viral Oncology, College of Chemistry and Life Science, Beijing University of Technology, Beijing 100124, China; lrh1217021@163.com; 2 Beijing Institute of Radiation Medicine, Beijing 100850, China; wangyuying0829@163.com (Y.W.); sunx0102@163.com (X.S.); zmxia22@163.com (Z.X.); tianying1977@126.com (Y.T.); chenbiqiong0501@163.com (B.C.); liusc118@163.com (S.L.)

**Keywords:** radiation-induced brain injury, ferroptosis, lipidomics, proteomics, *Ginkgo biloba*

## Abstract

Radiation-induced brain injury (RBI) is a severe complication of cranial radiotherapy that poses a significant clinical challenge due to a lack of effective treatments. Ferroptosis, an oxidative stress-driven cell death pathway, has been implicated in its pathogenesis. Here, we report that 75% ethanol (GBF-8), a novel subfraction isolated from male *Ginkgo biloba* flowers, confers significant protection against RBI. In a murine RBI model, GBF-8 administration restored cognitive function and alleviated neuroinflammation. We demonstrated that this neuroprotective effect is mechanistically linked to ferroptosis inhibition. Integrated proteomic and metabolomic profiling identified the Solute carrier family 7 member 11 (Slc7a11)–Eukaryotic Translation Initiation Factor 4E Binding Protein 1 (Eif4ebp1) axis as the primary target of GBF-8. This work not only establishes GBF-8 as a promising therapeutic candidate but also delineates a previously unrecognized regulatory axis for combating ferroptosis in RBI.

## 1. Introduction

Radiotherapy remains the cornerstone treatment for head and neck tumors and has substantially prolonged patient survival [1]. Nevertheless, even with advancements in high-precision radiotherapy technologies, exposure of adjacent or overlapping normal brain tissues is often unavoidable, making RBI a common and persistent complication among patients [2,3]. RBI manifests as progressive and irreversible cognitive decline, which not only limits the delivery of optimal radiation doses but also severely impairs quality of life [4]. In addition to its clinical relevance, the expanding application of radioactive materials in research, industry, and military settings further elevates the risk of unintended radiation exposure [5,6]. Currently, no effective therapies exist beyond symptomatic management, underscoring the urgent need for effective strategies to prevent or treat RBI.

Radiotherapy exerts its cytotoxicity largely through non-specific mechanisms, primarily the induction of ionizing radiation (IR)-generated Reactive Oxygen Species (ROS) and associated deoxyribonucleic acid (DNA) damage [7], with oxidative stress being a key driver of subsequent tissue injury. The central nervous system is especially vulnerable to such damage, a trait attributed to its high lipid content, elevated metabolic activity, and relatively weak antioxidant defenses [2,8]. Persistent oxidative stress amplifies this vulnerability, impairing neuronal self-repair and promoting cognitive decline [9,10]. This process can escalate to ferroptosis—an iron-catalyzed, regulated cell death pathway characterized by excessive lipid peroxidation(LPO) and ROS accumulation [11,12,13]. Accumulating evidence supports the role of IR in provoking ferroptosis *in vivo* [14,15,16], but the involved molecular networks are not fully delineated. Several pharmacological and natural agents have demonstrated the ability to modulate ferroptosis, highlighting their potential as radioprotective candidates against RBI [17,18].

Natural products with favorable safety profiles represent promising candidates for radioprotection. Among them, *Ginkgo biloba* L., a “living fossil” from the Quaternary glacial period, is endemic to China, which hosts over 70% of its global population [19]. Owing to its rich composition of bioactive compounds—including flavonoids, ginkgolides, and polysaccharides—it exhibits diverse pharmacological activities, including antioxidant and neuroprotective effects [20,21]. While ginkgo extracts have been reported to alleviate radiation-induced brain injury in rodents [22], the mechanisms underlying their radioprotective effects remain poorly elucidated. Notably, several ginkgo constituents, such as luteolin and bilobalide, have demonstrated anti-ferroptosis properties [23,24,25,26], suggesting a potential mechanism for their neuroprotective efficacy.

In this study, eight subfractions were isolated from ginkgo flowers and designated as GBF-1 to GBF-8, respectively. Among them, GBF-8 exhibited the most potent anti-radiation activity in vitro. Chemical profiling of GBF-8 using ultra-high-performance liquid chromatography coupled with quadrupole-Orbitrap high-resolution mass spectrometry (UHPLC-Q-Orbitrap HRMS) led to the identification of 125 compounds, including 76 flavonoids, 14 terpenoids, and other constituents. The therapeutic efficacy of GBF-8 was evaluated in a mouse model of RBI through behavioral tests, immunofluorescence, Western Blot (WB), and compound characterization. To elucidate its mechanism, we employed integrated proteomic and metabolomic analyses to investigate the underlying cellular mechanism by which GBF-8 inhibits ferroptosis. Our results indicate that GBF-8 alleviates ionizing radiation-induced damage by inhibiting oxidative stress and ferroptosis, underscoring its development as a natural treatment for RBI.

## 2. Materials and Methods

### 2.1. Plant Material

The fresh male flowers of ginkgo, collected in April from Tancheng, Shandong Province. The samples were then dried in an oven at 60 °C for 48 h to remove moisture while minimizing the degradation of bioactive components [20,27,28].

### 2.2. Chemicals and Reagents

All solvents, including petroleum ether, ethanol, chloroform, and ethyl acetate, were of analytical grade and purchased from Sinopharm Chemical Reagent Co., Ltd. (Shanghai, China). Sodium pentobarbital was obtained from Beijing Chemical Reagent Company (Beijing, China). Erastin and Ferrostatin-1 (Fer-1) were acquired from Selleck Chemicals (Houston, TX, USA).

Primary antibodies against the following proteins were purchased from Abcam (Waltham, MA, USA): Glial Fibrillary Acidic Protein (GFAP; cat# ab7260), xCT (Slc7a11; cat# ab307601) and Doublecortin (DCX; cat# ab18723). The Anti-GAPDH antibody (A19056) was obtained from ABclonal (Wuhan, China).

The following commercial kits were used according to the manufacturers’ protocols: Total Ribonucleic Acid (RNA) Extraction Kit (Shanghai Promega Biotech, Shanghai, China), Evo M-MLV One-Step RT-PCR Kit (Accurate Biotechnology, Hunan, China), Bicinchoninic Acid (BCA) Protein Assay Kit (Cowin Biotech, Jiangsu, China), Ferroptosis PCR Array (wc-mRNA0271-M; WCGENE Biotech, Shanghai, China), Calcein-AM/Propidium Iodide (Calcein-AM/PI) Double Staining Kit (Solarbio, Beijing, China), and GSH Assay Kit (Nanjing Jiancheng Bioengineering Institute, Nanjing, China).

### 2.3. Preparation of Active Components from Ginkgo Flowers

*Ginkgo biloba* flowers were extracted three times with 70% ethanol (2 h per cycle) under reflux. The combined extracts were concentrated under reduced pressure to yield ginkgo biloba flower crude extract. This extract was suspended in water and successively partitioned with petroleum ether, chloroform, ethyl acetate, and n-butanol. The n-butanol fraction was subsequently fractionated on an AB-8 macroporous adsorption resin column, eluted with a stepwise gradient of water, 25%, 50%, and 75% ethanol. Then, sequentially collect the fractions obtained after each purification step of Ginkgo flowers. Fractions from each purification step were collected, yielding the following purified fractions: ginkgo biloba flower total extract (GBF-1), Chloroform (GBF-2), Ethyl acetate (GBF-3), n-butanol (GBF-4), water (GBF-5), 25% ethanol (GBF-6), 50% ethanol (GBF-7), and 75% ethanol (GBF-8) fractions. All fractions were subjected to water removal via reduced-pressure distillation and subsequently stored as dry powders in a 4 °C refrigerator.

### 2.4. Chemical Composition Analysis by UHPLC-Q-Exactive MS

Chromatographic separation was performed on a Vanquish Flex UHPLC system (Thermo Scientific, Waltham, MA, USA) using an ACQUITY UPLC HSS T3 column (40 °C). The mobile phase consisted of 0.1% formic acid in water (A) and acetonitrile (B) at a flow rate of 0.3 mL/min. The gradient program was as follows: 0–1 min, 2% B; 1–14 min, 2–30% B; 14–25 min, 30–100% B; 25–28 min, 100% B.

Mass spectrometry was conducted on a Q Exactive hybrid quadrupole-Orbitrap mass spectrometer (Thermo Scientific) equipped with a HESI-II source. The source parameters were set as follows: spray voltage, 3.7 kV (positive) and 3.5 kV (negative); capillary temperature, 320 °C; sheath gas, 30 psi; auxiliary gas, 10 psi.

### 2.5. Pharmacodynamics Study on the Radiation-Induced Cognitive Impairment Model

#### 2.5.1. Cell Culture

PC-12 cells (a rat adrenal pheochromocytoma cell line) were maintained in DMEM supplemented with 5% fetal bovine serum, 10% horse serum, and 1% penicillin/streptomycin, and incubated at 37 °C in a humidified 5% CO_2_ atmosphere.

Anti-radiation assay: Cells were seeded at a density of 8 × 10^3^ cells/well and pretreated with GBF-8 for 2 h before exposure to ^60^Co γ-ray irradiation (8 Gy). Amifostine (Ami) served as the positive control.

#### 2.5.2. Animal Treatment

Specific pathogen-free (SPF) male C57BL/6J mice (8 weeks old, 20 ± 2 g) were purchased from SPF (Beijing) Biotechnology Co., Ltd. (Beijing, China) The animals were housed under standard conditions (24 ± 2 °C, 12 h light/dark cycle, 60% relative humidity) with free access to food and water. All experimental procedures were approved by the Animal Care and Use Committee of the Beijing Institute of Radiation Medicine and conducted in strict accordance with the institutional guidelines for the care and use of laboratory animals (Ethical approval No. IACUC-DWZX-2025-P519).

#### 2.5.3. Groups and Protocols

112 mice were randomly divided into four groups, with 28 mice in each group: (1) the Control group (healthy mice without irradiation); (2) the IR group, receiving purified water via intragastric gavage once daily for 10 days, covering the period from 7 days before to 3 days after irradiation; (3) the IR + GBF-8 group, receiving oral administration of GBF-8 at a dosage of 30 mg/kg once daily for 10 days, covering the period from 7 days before to 3 days after irradiation; (4) the IR +Ami group, receiving an intraperitoneal injection of Ami at a dosage of 150 mg/kg, administered the day before and 30 min before irradiation, administered the day before and 30 min before irradiation.

For irradiation, mice from all groups except the Control were exposed to 30 Gy of ^60^Co γ-rays directed to the whole head, using a lead shield to collimate the beam and confine the radiation field to the head. Control mice underwent the same procedures but without activation of the radiation source. This shielded irradiation approach ensured that vital functions such as eating and breathing were not compromised.

#### 2.5.4. Behavioral Evaluation

A series of behavioral tests was conducted to assess cognitive function and anxiety-like behavior through the Morris Water Maze (MWM), Open Field Test (OFT), and Novel Object Recognition (NOR) tests. To evaluate spatial learning and memory, eight mice per group were tested using the MWM. The acquisition phase (positioning navigation) was performed at 1, 3, 7, 14, and 21 days post-irradiation. Locomotor activity and anxiety-like behavior were assessed in eight mice per group using the OFT at 4 days post-irradiation. Short-term spatial memory and recognition were evaluated in six mice per group on day 4 post-irradiation using the NOR test.

#### 2.5.5. Biological Sampling and Processing

Blood samples were collected from the tail vein of eight mice in each group and analyzed with an automatic hematology analyzer (BC-2800Vet, Mindray, Shenzhen, China) for complete blood count determination. At 1, 4, 7, and 14 days post-irradiation, five mice were randomly chosen from each group for euthanasia. Body weight was measured and documented, followed by the collection of brain, thymus, and spleen tissues for further experimental assays.

#### 2.5.6. Immunofluorescence Staining for GFAP and DCX in the Mouse Hippocampus

For immunofluorescence staining, the left hemispheres of mouse brains, which were harvested as described in Section 2.5.5, were utilized. Specifically, three samples were randomly selected from each experimental group to ensure the representativeness of the results. Sequential 15 μm-thick coronal sections of the hippocampal region were prepared using a cryostat. After three 5-min washes with phosphate-buffered saline (PBS), sections were permeabilized with 0.3% Triton X-100 in PBS for 15 min and blocked with 10% bovine serum albumin for 1 h at room temperature. Sections were then incubated overnight at 4 °C with the following primary antibodies: anti-GFAP and anti-DCX. After three 8-min washes with PBS, sections were incubated with the corresponding secondary antibody (e.g., Cy3-conjugated donkey anti-rabbit IgG) for 1 h at room temperature. Images were acquired with either a fluorescence microscope or a laser-scanning confocal microscope and analyzed in a double-masked manner using ImageJ (v1.44p).

#### 2.5.7. qRT-PCR Analysis of Key Genes in the Mouse Brain Post-Irradiation

Total RNA was isolated from the hippocampus and reverse-transcribed. Gene expression analysis of ferroptosis-related markers was performed using qRT-PCR with three technical replicates. The 2^(−ΔΔCt) method was used for relative quantification, with *Gapdh* serving as the internal control.

#### 2.5.8. Western Blot Analysis of Key Proteins in the Mouse Brain Post-Irradiation

Western blotting was conducted as described previously [29]. Briefly, brain tissues were homogenized in RIPA lysis buffer supplemented with a protease inhibitor cocktail. Protein concentrations were determined using a BCA assay. Proteins were separated by SDS-PAGE on 12.5% polyacrylamide gels, transferred to PVDF membranes, and incubated overnight at 4 °C with the following primary antibodies: anti-Slc7a11, and anti-Gapdh. After incubation with Horseradish Peroxidase -conjugated secondary antibodies, protein bands were visualized using an enhanced chemiluminescence detection kit. Chemiluminescent signals were captured with a ChemiDoc^TM^ MP Imaging System (Bio-Rad Laboratories, Hercules, CA, USA). WB image quantification was performed using ImageJ software V1.4.4. The background of each blot was subtracted using the local background correction function. The integrated optical density (IOD) of target protein bands and internal reference bands (GAPDH) was measured separately. The relative expression level of the target protein was calculated as the ratio of the IOD value of the target protein to that of the internal reference protein. All WB experiments were repeated independently for three times, and three technical replicates were set in each experiment to ensure the reliability of the quantitative results.

### 2.6. Mechanistic Study on the Role of GBF-8 in Regulating Ferroptosis In Vitro

#### 2.6.1. Cell Culture and Assessment of Ferroptosis Markers

PC-12 cells were maintained in DMEM supplemented with 5% fetal bovine serum, 10% horse serum, and 1% penicillin/streptomycin, and incubated at 37 °C in a humidified 5% CO_2_ atmosphere.

Anti-ferroptosis assay: Cells were seeded at 8 × 10^3^ cells/well in 100 μL of complete medium into 96-well plates. Except for the standard control, all other groups were treated with 10 μM Erastin to induce ferroptosis. Treatment groups received GBF-8 at final concentrations of 25–100 μg/mL, while Fer-1 was used as a positive control.

After 24 h of incubation, cell viability was assessed using the CCK-8 assay. The culture medium was replaced with fresh medium containing 10% CCK-8 reagent, and the plate was incubated for 3 h, after which absorbance was measured at 450 nm using a microplate reader. Detailed methods for transmission electron microscopy, Live/Dead Staining, and assays for LPO, ROS, Fe^2+^, and GSH are provided in the Supplementary Materials.

#### 2.6.2. Targeted Lipidomics by Mass Spectrometry

Targeted lipidomic analysis of PC-12 cells was performed based on established methodologies. The procedure included lipid extraction and quantification using ultra-high-performance liquid chromatography-tandem mass spectrometry (UPLC-MS/MS; Beijing HeXin Biotech Co., Ltd., Beijing, China). Complete methodological details are available in the Supplementary Material.

#### 2.6.3. Proteomic Analysis of GBF-8-Regulated Key Proteins in Ferroptosis

Label-free quantitative proteomics was applied to compare protein expression between control and Erastin-induced PC-12 cells. In addition, parallel reaction monitoring (PRM) was used to validate the regulatory effects of GBF-8 on Erastin-induced differential protein expression.

Protein identification was conducted using UPLC-MS/MS (Beijing Qinglian Biotech Co., Ltd., Beijing, China). Data were processed with Proteome Discoverer (v2.4) and Skyline software (v23.1).

### 2.7. Statistical Analysis

All experiments were performed in triplicate. Data in bar graphs are presented as the mean ± standard deviation. Differences among multiple groups were assessed using one-way analysis of variance (ANOVA), with *p*-values < 0.05 considered statistically significant (* *p* < 0.05, ** *p* < 0.01, *** *p* < 0.001). All statistical analyses were performed using GraphPad Prism software (v9.0.0).

## 3. Results

### 3.1. Preparation and Screening of Anti-Radiation Ginkgo Flower Fractions

**Figure 1:**
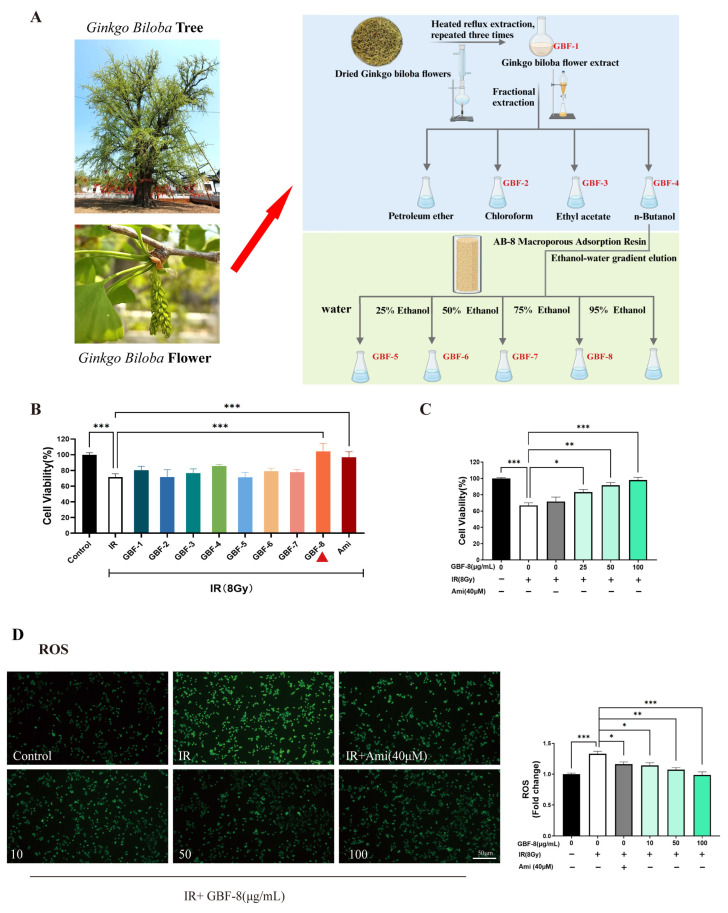
Radioprotective effect of GBF-8 on PC12 cells. (**A**) The preparation of different components from *Ginkgo biloba* flowers (**B**) Radioprotective effect of irradiated PC12 cells treated with various GBF extracts (100 μg/mL). (**C**) Dose-dependent effect of GBF-8 (25–100 μg/mL) on the viability of irradiated cells. (**D**) GBF-8 (10–100 μg/mL) suppresses radiation-induced ROS generation. One-way ANOVA analyzed data; compared with the IR group, * *p* < 0.05, ** *p* < 0.01, and *** *p* < 0.001.

The preparation of different components from *Ginkgo biloba* flowers is illustrated in Figure 1A. The radioprotective activity of each *Ginkgo biloba* flower extract was evaluated in PC12 cells irradiated with 8 Gy of ^60^Co γ-rays. Cells were treated with GBF-1 to GBF-8 at a concentration of 100 μg/mL, using 40 μM Ami as a positive control. The CCK-8 assay determined cell viability.

At 24 h post-irradiation, PC12 cell viability decreased to approximately 70%. Treatment with the GBF-8 or Ami significantly restored cell viability. Although the total extract, GBF-4, and GBF-6 also showed a tendency to increase viability, the effects were not statistically significant (Figure 1B). Furthermore, GBF-8 increased cell viability in a dose-dependent manner at concentrations ranging from 25 to 100 μg/mL (Figure 1C). At 100 μg/mL, GBF-8 restored viability to levels comparable to those of the non-irradiated control.

IR induces cellular damage primarily through DNA strand breaks and ROS accumulation [30]. Excessive ROS oxidatively damages key cellular biomolecules, including proteins, lipids, and nucleic acids, ultimately leading to cell death. Using the fluorescent probe DCFH-DA to assess intracellular ROS levels, we observed a marked increase in ROS in irradiated PC12 cells, as indicated by higher green fluorescence intensity than in the control group (Figure 1D). Treatment with GBF-8 significantly suppressed radiation-induced ROS production, consistent with the improved cell viability observed in the CCK-8 assay. These results suggest that the radioprotective effect of GBF-8 is at least partially mediated by attenuating radiation-induced ROS accumulation.

### 3.2. Chemical Composition Analysis of GBF-8

**Figure 2:**
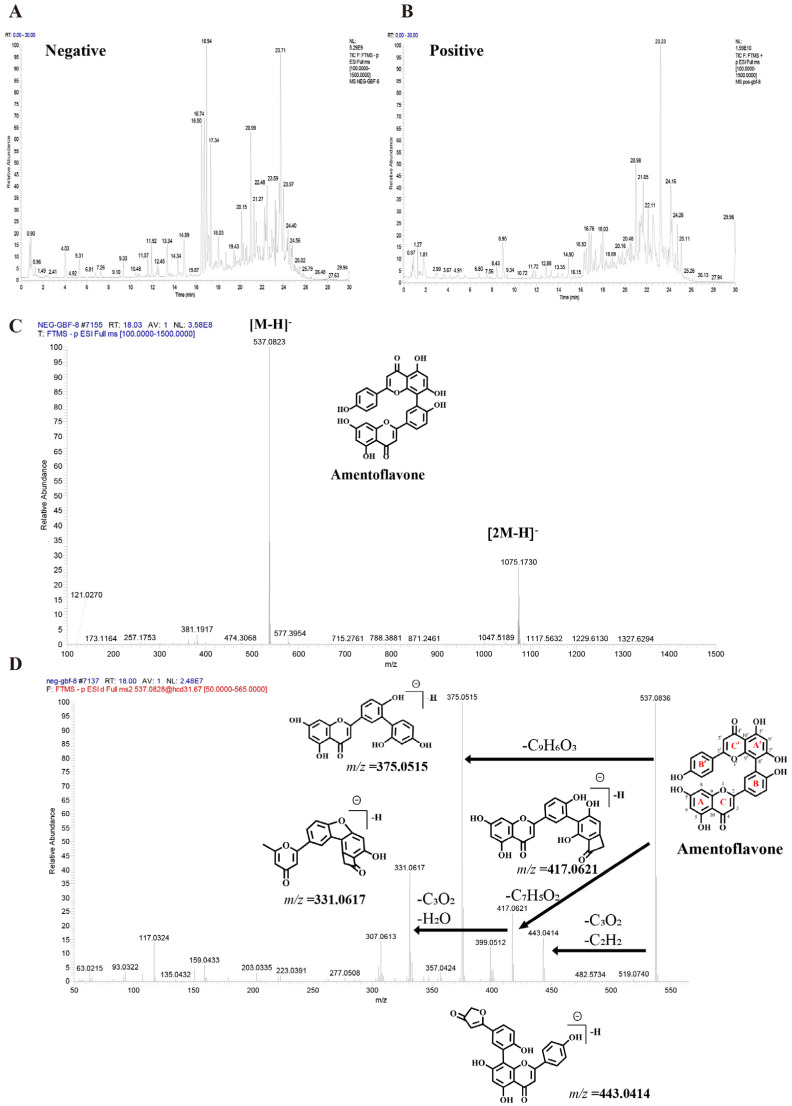
Base peak chromatograms of the GBF-8 extract obtained by UHPLC-Q-Orbitrap HRMS. (**A**) Negative and (**B**) positive electrospray ionization modes. (**C**) MS^1^ spectrum of Amentoflavone. (**D**) MS^2^ spectrum of Amentoflavone.

UHPLC-Q-Orbitrap HRMS characterized the chemical profile of GBF-8 in positive and negative ion modes (Figure 2). Compounds were identified by matching against the TCM Pro 2.0 reference database and a curated theoretical database, using a multi-dimensional strategy that considered retention time, precursor mass accuracy, MS/MS fragmentation, isotope pattern, and signal intensity.

Figure 2C,D illustrates the identification process of the biflavonoid Amentoflavone from GBF-8. In the negative-ion mode total ion chromatogram of GBF-8, two characteristic peaks at a retention time of 18.03 min corresponded to the [M-H]^−^ ion (*m*/*z* 537.0823) and [2M-H]^−^ ion (*m*/*z* 1075.1730), respectively. Based on these data, the molecular weight of the compound was preliminarily determined to be 538.0903 (Figure 2C). Subsequently, collision-induced dissociation was performed using [M-H]^−^ (*m*/*z* 537.0823) as the precursor ion. MS^2^ analysis revealed major fragment ions at *m*/*z* 375.0515, 443.0414, 417.0621, and 331.0617, with relative abundances of 100%, 14.14%, 24.54%, and 38.62%, respectively. It was hypothesized that, in the MS^2^ spectrum, the [M-H]^−^ ion of this compound at *m*/*z* 537.0823 underwent fragmentation to yield a series of characteristic product ions: the [M-H-C_6_H_6_O]^−^ ion at *m*/*z* 443.0414, the [M-H-C_7_H_4_O_2_]^−^ ion at *m*/*z* 417.0621, the [M-H-C_9_H_6_O_3_]^−^ ion at *m*/*z* 375.0515, and the [M−H−C_10_H_6_O_5_]^−^ ion at *m*/*z* 331.0617.The fragment ion at *m*/*z* 375.0515 likely originated from cleavage of the C′-ring 9′,10′-bond with the loss of a C_9_H_6_O_3_ moiety, consistent with characteristic fragmentation pathways of biflavonoids. The fragment at *m*/*z* 443.0414 is proposed to result from McLafferty rearrangement of the A-ring followed by loss of C_3_O_2_ and subsequent C′-ring cleavage. Comparison with published literature confirmed that among fragmentation patterns align with those reported for Amentoflavone, thereby confirming the identity of this compound as Amentoflavone [31].

Low-confidence matches were excluded, leading to the identification of 125 compounds [32,33,34,35,36,37,38,39,40,41,42,43,44,45,46,47,48,49,50,51,52,53,54,55,56,57,58,59,60,61,62,63,64,65,66,67,68,69,70,71,72,73,74,75,76,77,78,79,80,81,82,83,84,85,86,87,88,89,90,91,92,93,94] (see Table S2 in the Supplementary Materials for complete details). These included 76 flavonoids, 14 terpenoids, 8 fatty acids, 6 phenolic acids, 5 alkaloids, and minor constituents such as phenylpropanoids, steroids, and amino acids.

### 3.3. GBF-8 Ameliorates Radiation-Induced Cognitive Impairment in Mice

**Figure 3:**
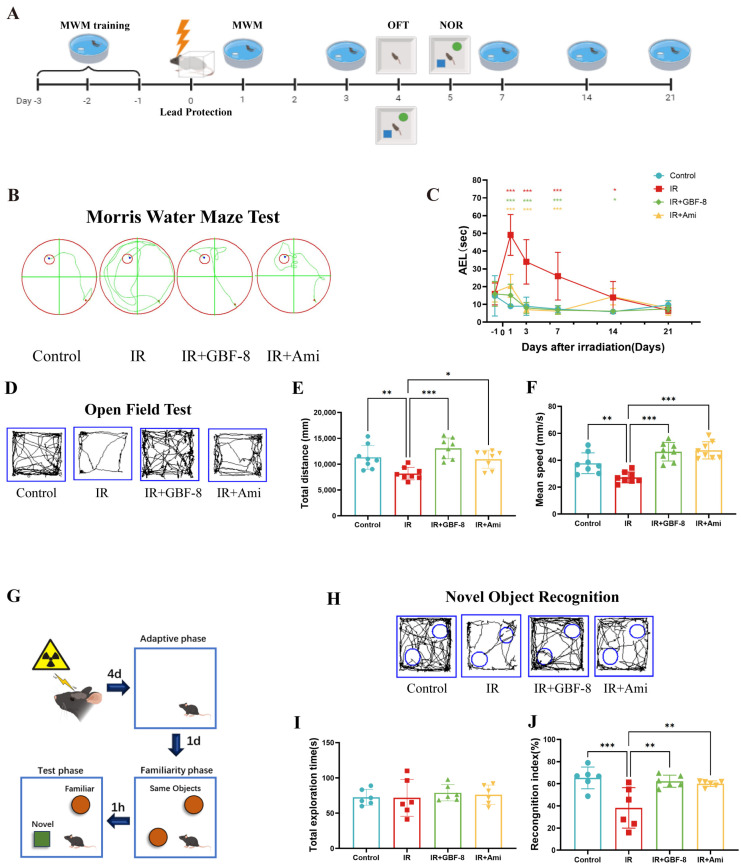
GBF-8 ameliorates radiation-induced cognitive deficits in mice. (**A**) Experimental timeline for behavioral tests. (**B**,**C**) The MWM test assessed spatial learning and memory (*n* = 8). (**B**) Representative escape paths. (**C**) Average escape latency across the 21-day testing period. (**D**–**F**) OFT for locomotor activity and anxiety-like behavior (*n* = 8). (**D**) Representative movement trajectories. (**E**) Total distance traveled. (**F**) Average moving speed. (**G**–**J**) NOR test for short-term memory (*n* = 6). (**G**) Schematic of the experimental setup. (**H**) Typical exploration paths. (**I**) Total object exploration time. (**J**) Recognition index calculation. One-way ANOVA analyzed data; compared with the IR group, * *p* < 0.05, ** *p* < 0.01, and *** *p* < 0.001.

The experimental timeline is shown in Figure 3A. Spatial learning and memory were assessed using the MWM test. Mice in the IR group exhibited significantly prolonged escape latency compared to the control group (Figure 3B,C), indicating substantial cognitive impairment. Treatment with GBF-8 (IR+GBF-8 group) significantly rescued this deficit, as shown by markedly shortened escape latency. This restorative effect was consistent from days 1 to 14 post-irradiation. By day 21, the escape latency of the IR group had recovered to a level comparable to that of the controls, suggesting a time-dependent recovery from radiation-induced cognitive impairment (Figure 3C).

The OFT was used to evaluate anxiety-like behavior and locomotor activity. Mice in the IR group exhibited significant reductions in both total distance traveled and average velocity, indicating radiation-induced hypoactivity and anxiety-like behavior (Figure 3D–F). GBF-8 treatment significantly restored both total distance traveled and average speed in irradiated mice, with efficacy comparable to that of the positive control drug. These results indicate that GBF-8 effectively reverses radiation-induced deficits in exploratory behavior.

The NOR test assesses short-term memory, with the cognitive index (time exploring novel object/total exploration time) serving as the primary metric (Figure 3G). Compared to controls, mice in the IR group showed a significantly reduced cognitive index, indicating impaired recognition memory (Figure 3G–J). GBF-8 treatment significantly restored this index, demonstrating its efficacy in ameliorating radiation-induced memory deficits.

### 3.4. GBF-8 Improves Multiple Indices After Radiation Exposure

**Figure 4:**
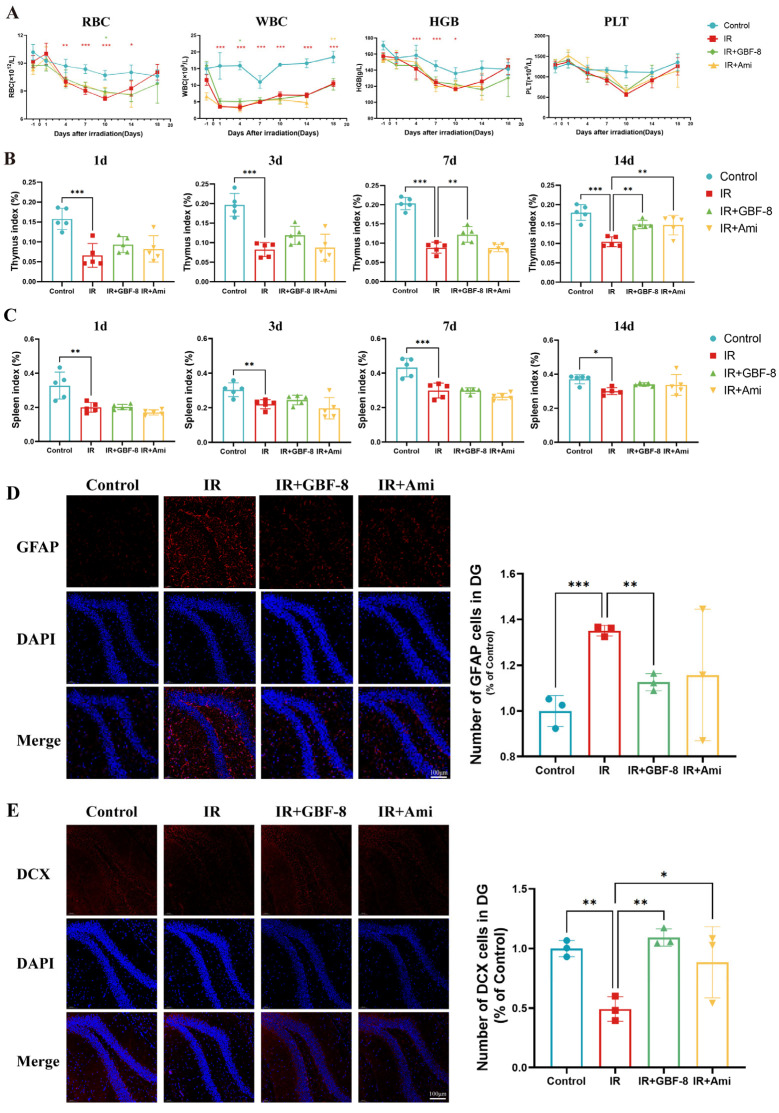
GBF-8 mitigates radiation-induced hematological and histological injury in mice. (**A**) Dynamic changes in peripheral blood cell counts following irradiation (*n* = 8). (**B**,**C**) Thymus (**B**) and spleen (**C**) indices (organ weight/body weight ratio) at indicated time points post-irradiation (*n* = 5). (**D**) Immunofluorescence analysis of astrocyte activation in the hippocampus (*n* = 3). (**E**) Immunofluorescence analysis of neurogenesis in the hippocampus (*n* = 3). One-way ANOVA analyzed data; compared with the IR group, * *p* < 0.05, ** *p* < 0.01, and *** *p* < 0.001.

Bone marrow, a highly radiosensitive hematopoietic tissue, is vulnerable to radiation-induced damage. IR directly impairs hematopoietic stem cells, leading to hematopoietic dysfunction [95]. In this study, localized cerebral irradiation significantly altered peripheral blood cell counts in mice (Figure 4A). Red blood cell (RBC) counts declined after irradiation, reaching a nadir on day 10 before recovering by day 18. RBC counts in the IR group were significantly lower than in the Control group from day 4 to day 14, but were partially restored in the IR+GBF-8 group on day 10. White blood cell (WBC) counts dropped sharply post-irradiation, with the lowest levels on day 1 and sustained suppression thereafter. WBC counts in the IR group were significantly lower than in the Control group from day 1 to day 18, while the IR+GBF-8 group showed significant recovery by day 7. Hemoglobin (HGB) levels gradually decreased, reaching the lowest point on day 10 before rebounding by day 18. HGB in the IR group was significantly lower than in Controls on days 1, 4, and 7. Platelet (PLT) counts progressively declined, with the lowest values on day 10. PLT counts were significantly lower in the IR group than in Controls on days 7 and 10, but were significantly higher in the IR+GBF-8 group than in the IR group on day 7. By day 18, PLT counts returned to baseline across all groups. These time-dependent restorative effects indicate that GBF-8 promotes hematopoietic recovery after radiation injury.

Thymus and spleen indices were measured on days 1 to 14 post-irradiation (Figure 4B,C). The thymus index in the IR group was significantly reduced on days 1 to 14. GBF-8 treatment significantly restored the thymus index on days 7 to 14. (Figure 4B). Although irradiation also reduced the spleen index from days 1 to 14, GBF-8 only partially attenuated this decline without reaching statistical significance (Figure 4C). These results indicate that GBF-8 provides substantial protection against radiation-induced thymic damage during the critical recovery period.

The hippocampus was selected for immunofluorescence analysis due to its critical roles in learning and memory, and its exceptional radiosensitivity. We assessed the hippocampal response at day 4 post-irradiation. GFAP, a marker of astrocyte activation, was significantly upregulated in the dentate gyrus following irradiation, indicating astrogliosis [96]. GBF-8 treatment effectively suppressed radiation-induced GFAP activation and significantly enhanced DCX expression, a marker of neuronal precursor differentiation, suggesting its potential to promote neurogenesis after radiation injury.

### 3.5. Ferroptosis in Radiation-Induced Brain Injury and the Potential Regulatory Role of GBF-8

Ferroptosis, an iron-dependent form of regulated cell death driven by LPO, is increasingly implicated in radiation-induced tissue injury. A growing consensus suggests that ferroptosis contributes to RBI, sharing pathological features, notably iron accumulation and LPO, with other brain disorders [97,98]. To test this hypothesis, we assessed key ferroptosis markers in brain tissue from irradiated mice using qPCR and WB.

**Figure 5:**
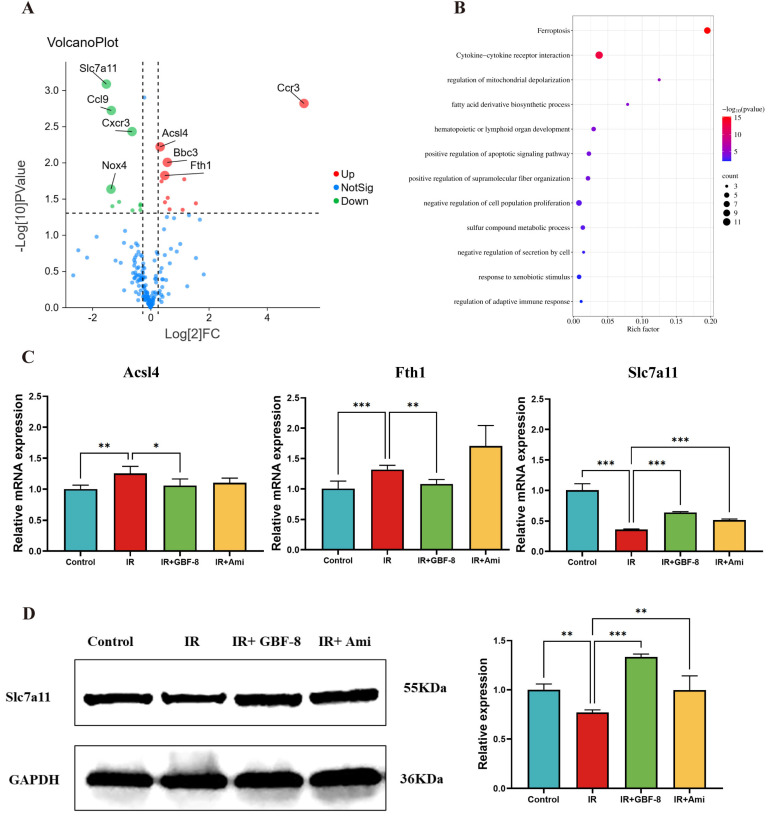
GBF-8 modulates irradiation-induced gene and protein expression in mouse brain tissue. (**A**) Volcano plot of differentially expressed gene between Control and IR group. Red points: upregulated proteins; green points: downregulated proteins. (**B**) KEGG pathway enrichment (Control vs. IR). Pathways are sorted by enrichment ratio; dot size reflects the number of gene. (**C**) GBF-8 regulates the mRNA expression of Slc7a11, Acsl4, and Fth1 following IR (*n* = 3). (**D**) Slc7a11 protein levels in brain tissues. One-way ANOVA and *t*-test were used to analyze the data; * *p* < 0.05, ** *p* < 0.01, and *** *p* < 0.001 indicate differences compared with the IR group.

A total of 175 genes associated with IR and involved in multiple pathways, including oxidative stress, inflammation, ferroptosis, and apoptosis, were selected for analysis of differential expression between the Control and IR groups (Figure 5A). The results revealed that 27 genes were differentially expressed in mouse brain tissue after irradiation. Among these, 16 were significantly upregulate, including genes *Arachidonate 12-Lipoxygenase (Alox12)*, *Acyl-CoA Synthetase Long-Chain Family Member 4 (Acsl4), Aldo-Keto Reductase Family 1 Member C1 (Akr1c1), BCL2 Binding Component 3 (Bbc3), B-Rapidly Accelerated Fibrosarcoma (Braf), C-C Motif Chemokine Ligand 12 (Ccl12), C-C Motif Chemokine Receptor 3 (Ccr3), Ferritin Heavy Chain 1 (Fth1), Glutamate-Cysteine Ligase Catalytic Subunit (Gclc), Interleukin 15 (Il15), Iron Responsive Element Binding Protein 2 (Ireb2), Lymphotoxin Alpha (Lta), Nuclear Factor, Erythroid 2 Like 2 (Nfe2l2), Pannexin 2 (Panx2), Solute Carrier Family 40 Member 1 (Slc40a1),* and *Six-Transmembrane Epithelial Antigen of the Prostate 3 (Steap3).* While 11 genes were significantly downregulated, including *ADP Ribosylation Factor 6 (Arf6), C-C Motif Chemokine Ligand 22 (Ccl22), C-C Motif Chemokine Ligand 6 (Ccl6), C-C Motif Chemokine Ligand 9 (Ccl9), CDGSH Iron Sulfur Domain 2 (Cisd2), C-X-C Motif Chemokine Receptor 3 (Cxcr3), Glutaminase 2 (Gls2), Interleukin 10 Receptor Subunit Alpha (Il10ra), Nuclear Receptor Coactivator 4 (Ncoa4), NADPH Oxidase 4 (Nox4), Slc7a11.* Kyoto Encyclopedia of Genes and Genomes (KEGG) pathway analysis indicated that these differentially expressed genes were predominantly enriched in the ferroptosis pathway (Figure 5B). In addition, GBF-8 treatment modulated the expression of key ferroptosis-related genes (Acsl4, Fth1, and Slc7a11), effectively counteracting their dysregulation induced by ionizing radiation (Figure 5C).

To further investigate the regulatory role of GBF-8 in radiation-induced brain injury, we assessed Slc7a11 protein expression by WB. Results showed a significant reduction in Slc7a11 expression at 7 days post-irradiation (*p* < 0.05). In contrast, GBF-8 treatment substantially restored Slc7a11 levels (*p* < 0.001), indicating a role for GBF-8 in counteracting ferroptosis after radiation exposure (Figure 5D).

### 3.6. GBF-8 Attenuates Erastin-Induced Ferroptosis in PC12 Cells

Building on previous findings that irradiation induces ferroptosis in a mouse model of RBI and that GBF-8 alleviates associated cognitive and hippocampal damage, we further investigated its anti-ferroptotic mechanism using PC12 cells.

**Figure 6:**
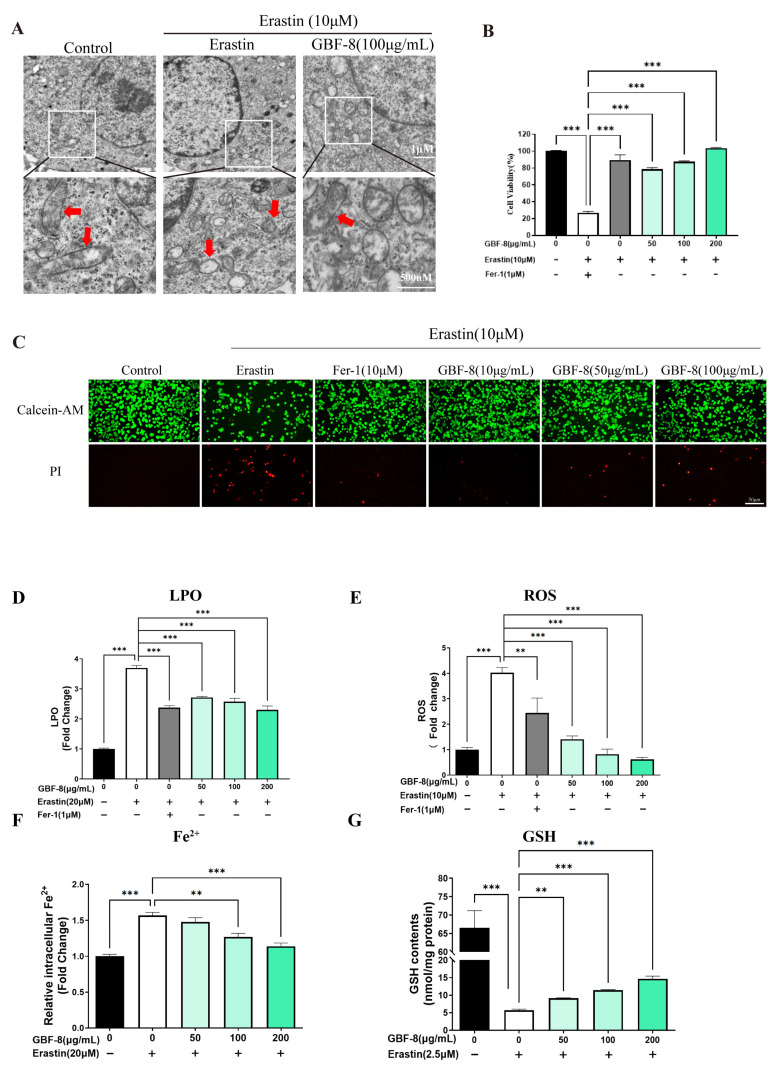
GBF-8 attenuates Erastin-induced ferroptosis in PC12 cells. (**A**) TEM observation of mitochondria in PC12 cells. (Mitochondria were highlighted with red arrows.) (**B**) Cell viability measured by CCK-8. (**C**) Cell viability assessed by Calcein-AM (live, green)/PI (dead, red) staining. (**D**–**G**) Intracellular LPO (**D**), ROS (**E**), Fe^2+^ (**F**), and GSH (**G**) levels. Data are presented as the mean ± SD. ** *p* < 0.01, *** *p* < 0.001 indicate differences compared with the Erastin model group.

Transmission electron microscopy (TEM) revealed that Erastin (10 μM) induced characteristic mitochondrial alterations in PC12 cells, including shrinkage, increased membrane density, and reduced cristae, consistent with ferroptotic morphology (Figure 6A). The CCK-8 assay showed that Erastin significantly decreased cell viability, while GBF-8 treatment dose-dependently restored viability at concentrations of 50–200 μg/mL (Figure 6B).

Calcein-AM/PI staining further confirmed the protective effect of GBF-8 against Erastin-induced ferroptosis. As shown in Figure 6C, Erastin-treated PC12 cells exhibited reduced green fluorescence (live cells) and increased red fluorescence (dead cells). In contrast, co-treatment with GBF-8 markedly increased the number of live cells and decreased the number of cells killed. These findings are consistent with the viability recovery observed in the CCK-8 assay (Figure 6B).

Based on the anti-ferroptotic activity of GBF-8, we further assessed its effects on key ferroptosis hallmarks (Figure 6D–G). Erastin treatment markedly increased LPO in PC12 cells, which GBF-8 significantly suppressed at concentrations of 50–200 μg/mL (Figure 6D). We also measured intracellular ROS and observed a pronounced Erastin-induced increase, which was effectively inhibited by GBF-8 (50–200 μg/mL) (Figure 6E).

Flow cytometric analysis revealed that Erastin elevated intracellular Fe^2+^ levels, an effect significantly attenuated by GBF-8 (50–200 μg/mL) (Figure 6F). Given that certain antioxidants act by chelating iron or suppressing iron-mediated redox cycling, we also evaluated GSH levels. Erastin-induced GSH depletion was substantially reversed by GBF-8 co-treatment (50–200 μg/mL; Figure 6G). Together, these results indicate that GBF-8 inhibits Erastin-induced ferroptosis by attenuating LPO, ROS production, and Fe^2+^ accumulation, while restoring GSH content.

### 3.7. GBF-8 Modulates Lipid Metabolism in Erastin-Treated PC12 Cells

**Figure 7:**
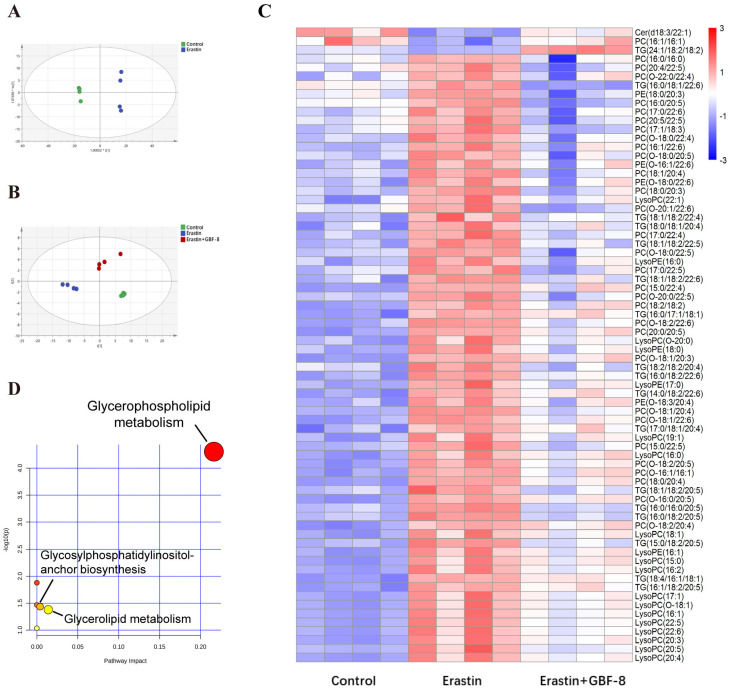
GBF-8 modulates lipid metabolism in Erastin-induced ferroptosis of PC12 cells. (**A**) PCA score plot (*n* = 4). (**B**) PLS-DA score plot. (**C**) Heatmap of 72 differentially expressed lipid metabolites. Blue and red indicate downregulation and upregulation, respectively. (**D**) Pathway enrichment analysis.

Lipids represent more than 50% of the dry weight of brain tissue and are critically involved in lipid metabolism pathways central to ferroptosis. To investigate lipidomic alterations, PC12 cells were allocated into three experimental groups: Control and Erastin group. Principal Component Analysis (PCA) revealed significant differences between these two groups (Figure 7A). Partial least squares-discriminant analysis (PLS-DA) conducted in the Control, Erastin, and Erastin+GBF-8 groups yielded high model validity confirming robust group discrimination (Figure 7B). In both positive and negative electrospray ionization modes, a total of 260 lipid metabolites were identified as differentially expressed in Erastin-treated cells (VIP > 1, *p* < 0.05), indicating substantial lipid metabolic disruption during ferroptosis. A comparative analysis of the GBF-8 treatment group identified 72 lipid species that were consistently altered. Among these, ceramide (Cer, d18:3/22:1), phosphatidylcholine (PC, 16:1/16:1), and triglyceride (TG, 24:1/18:2/18:2) were downregulated by Erastin, while the upregulated lipids included: 30 phosphatidylcholines (PCs), 19 lysophosphatidylcholines (LysoPCs), 16 triglycerides (TGs), and 4 phosphatidylethanolamines (PEs). GBF-8 treatment effectively restored the expression of these altered lipid metabolites toward normal levels (Figure 7C). Further pathway enrichment analysis revealed significant alterations primarily concentrated within the glycerophospholipid metabolism pathway (Figure 7D).

### 3.8. Proteomic Profiling of Erastin-Induced Ferroptosis in PC12 Cells

**Figure 8:**
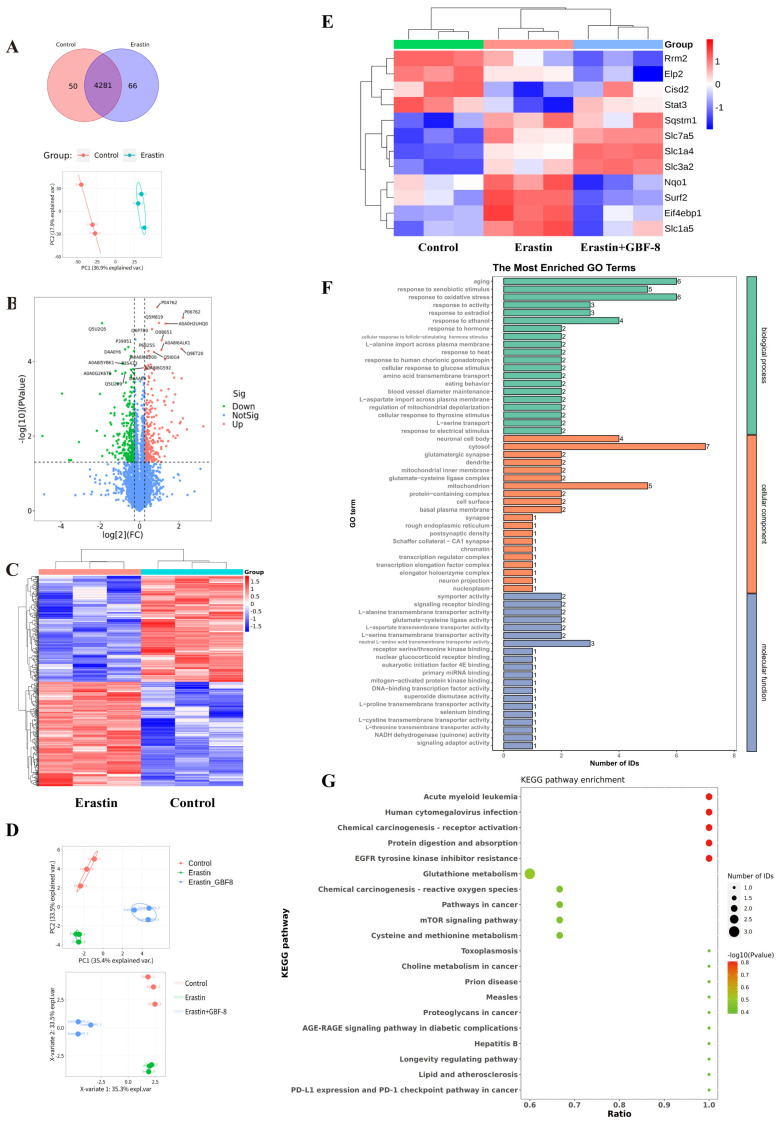
Proteomic analysis reveals GBF-8-mediated regulation of protein expression during Erastin-induced ferroptosis in PC12 cells. (**A**) Venn diagram and PCA score plot. (**B**) Volcano plot of differentially expressed proteins. Red points: upregulated proteins; green points: downregulated proteins. (**C**) Heatmap of 530 differentially expressed proteins identified by label-free untargeted proteomics. Blue and red indicate downregulation and upregulation, respectively. (**D**,**E**) Treatment groups: Control (DMSO), Erastin (5 μM), or Erastin + 200 μg/mL GBF-8 for 24 h (*n* = 3). (**D**) PCA score plot and PLS-DA score plot from targeted Proteomics. (**E**) Heatmap of differentially expressed proteins among Control, Erastin, and Erastin+GBF-8 groups from PRM-based targeted proteomics. (**F**) GO enrichment analysis (Erastin vs. Erastin+GBF-8). (**G**) KEGG pathway enrichment (Erastin vs. Erastin+GBF-8). Pathways are sorted by enrichment ratio; dot size reflects the number of proteins.

To elucidate the anti-ferroptotic mechanism of GBF-8, label-free quantitative proteomics was performed on PC12 cells treated for 24 h with DMSO (Control) or Erastin. Among 4281 consistently quantified proteins, PCA showed clear separation between groups (Figure 8A). Using criteria of fold change > 1.2 or <1/1.2 with *p* < 0.05, we identified 530 significantly altered proteins (263 upregulated, 267 downregulated) in Erastin-treated cells (Figure 8B). Hierarchical clustering revealed distinct expression patterns among these proteins, with color intensity indicating relative abundance (Figure 8C), demonstrating that Erastin substantially remodels the proteomic profile of PC12 cells during ferroptosis.

Candidate proteins were selected based on both significant differential expression (fold change > 1.2 or <1/1.2, *p* < 0.05) and prior association with ferroptosis. Subsequently, PRM was employed to validate 29 candidates in PC12 cells subjected to Erastin with or without GBF-8 (Figure 8D, E). This analysis confirmed 12 proteins significantly altered by Erastin (8 upregulated, 4 downregulated). Notably, GBF-8 treatment reversed Erastin-induced changes in several key proteins: it downregulated NAD(P)H quinone dehydrogenase 1 (Nqo1), Eif4ebp1, solute carrier family 1 member 5 (Slc1a5), and SUMO2-conjugating enzyme UBC9-interacting protein (Surf2), while upregulating signal transducer and activator of transcription 3 (Stat3). These PRM-validated targets represent potential mediators of the anti-ferroptotic activity of GBF-8 in PC12 cells.

Gene Ontology (GO) enrichment analysis of differentially expressed proteins between Erastin+GBF-8 and Erastin groups revealed functionally relevant terms across three categories (Figure 8F). In biological processes, “aging” and “response to xenobiotic stimulus” were most significantly enriched, suggesting their potential involvement in the cellular response to GBF-8. Additionally, “response to oxidative stress” was notably enriched, supporting a role for redox—and activity-dependent pathways in GBF-8-mediated modulation of ferroptosis. Cellular component analysis identified “neuronal cell body” and “cytosol” as predominant terms, indicating substantial proteomic alterations in neuronal compartments. Within molecular function, the most significantly enriched terms included “L-alanine transmembrane transporter activity” and “glutamate-cysteine ligase activity,” implicating amino acid transport and glutathione synthesis in the anti-ferroptotic mechanism of GBF-8.

KEGG pathway enrichment analysis of the proteomic data revealed diverse biological processes potentially modulated by GBF-8 (Figure 8G). Significantly enriched pathways spanned multiple functional domains, including cancer-related pathways (Pathways in cancer), metabolic pathways (Glutathione metabolism), and signaling cascades (mTOR signaling pathway). Notably, “Glutathione metabolism” showed a high ratio and strong statistical significance, consistent with the known antioxidant and ferroptosis-modulating effects of GBF-8. Enrichment of the “mTOR signaling pathway” further supports its involvement in cellular growth and stress responses, aligning with observed changes in protein synthesis-related factors. Together, these findings highlight the multi-pathway regulatory role of GBF-8 and provide a systems-level perspective for understanding its bioactivity against radiation-induced injury and ferroptosis.

## 4. Discussion and Conclusions

In this study, we first subjected *Ginkgo biloba* male flowers to heat reflux extraction, followed by fractionation of the total extract. Ultimately, eight subfractions (GBF-1 to GBF-8) were obtained. *In vitro* anti-radiation activity screening revealed that GBF-8 exhibited the most potent anti-radiation activity, making it the optimal active subfraction. Subsequently, we identified the chemical constituents of GBF-8. GBF-8 exhibited protective effects in aRBI mouse model by accelerating recovery, alleviating hippocampal damage, mitigating fear, anxiety, and cognitive impairment, and promoting immune system restoration, likely through modulation of the ferroptosis pathway.

GBF-8 contains multiple compounds—such as flavonoids (e.g., quercetin, baicalin, luteolin) and terpenoids (e.g., ginkgolides, bilobalide)—known to possess antioxidant, anti-inflammatory, and iron-chelating activities, which may contribute to its ability to regulate ferroptosis and alleviate radiation injury [99,100]. These compositional features support the observed anti-ferroptotic and radioprotective effects of GBF-8.

The mouse model of RBI was established via local irradiation of the mouse brain with a 30 Gy ^60^Co γ-ray. Behavioral assay results indicated that IR impaired spatial learning and memory abilities in mice, while GBF-8 significantly alleviated such radiation-induced deficits in spatial learning and memory. However, the OFT in this study was conducted with a sample size of only six mice per group, which imposes certain limitations on the conclusions regarding radiation-induced memory impairment drawn herein. In subsequent studies, we will expand the experimental sample size to further investigate the ameliorative effect of GBF-8 on memory function in mice. Additionally, our results demonstrated that GBF-8 enhanced immune function in RBI mice by improving peripheral blood cell counts, thymus index, and spleen index.

Transcriptomic analysis of irradiated mouse brain tissue revealed dysregulation of genes related to oxidative stress, iron metabolism, and inflammation. Upregulation of genes, including Acsl4—implicated in LPO—and inflammatory mediators (Ccl12, Ccr3, Il15) indicated enhanced lipid damage and localized neuroinflammation [101,102]. Conversely, downregulation of Slc7a11 likely impaired antioxidant defense, exacerbating oxidative stress, while reduced expression of Cisd2 and Ncoa4 further disrupted iron homeostasis. Together, these changes suggest that radiation triggers ferroptosis through integrated pathways involving LPO, iron dysregulation, and compromised antioxidant capacity.

Our lipidomic profiling identified triglycerides (TGs) and PCs as the major classes of differentially expressed lipids. Under high-iron conditions, polyunsaturated fatty acids (PUFAs) released from TGs can undergo peroxidation, resulting in membrane damage and cell death [103]. PCs contribute to ferroptosis by peroxidizing their PUFA chains, thereby compromising membrane integrity [104]. GBF-8 treatment reversed the Erastin-induced upregulation of TGs and PCs, suggesting a membrane-stabilizing effect that may attenuate ferroptosis [103]. Furthermore, PUFA metabolism generates ROS, and excess ROS can induce oxidative stress and trigger ferroptosis. Specific lipids, including TGs and PCs, may also promote ferroptosis by impairing intracellular antioxidant defenses [103]. Importantly, GBF-8 restored the composition of PUFAs and PUFA-containing phospholipids disrupted by Erastin. These results indicate that GBF-8 protects against ferroptosis in PC12 cells, at least in part, by enhancing cellular antioxidant capacity and rectifying lipid metabolic dysregulation.

Proteomic analysis highlighted oxidative stress as a central pathway modulated by GBF-8 in PC12 cells. *In vivo* studies confirmed that GBF-8 rescues irradiation-induced mRNA dysregulation of Slc7a11, Acsl4, and Fth1—key genes involved in glutathione synthesis, LPO, and iron sequestration, respectively [105,106]. KEGG pathway enrichment further identified the mTOR signaling cascade as pivotal for GBF-8 activity. Notably, GBF-8 altered phosphorylation of Eif4ebp1 (4EBP1), an mTORC1 substrate that regulates cap-dependent translation. Given the established role of mTOR/4EBP1 signaling in upregulating Slc7a11 and suppressing ferroptosis, we propose that GBF-8 activates the mTOR/4EBP1 axis to enhance Slc7a11 expression, thereby conferring cytoprotection against ferroptosis [107].

**Figure 9:**
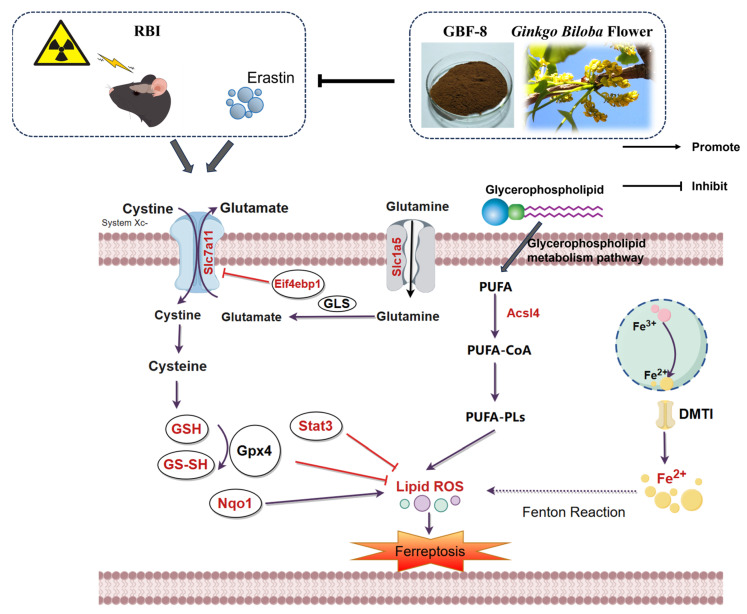
The potential mechanism of irradiation-induced ferroptosis and regulatory targets of GBF-8. Key pathway components modulated by GBF-8 are highlighted in red (By http://www.figdraw.com, accessed on 4 January 2026).

Collectively, these results demonstrate that GBF-8 exerts anti-ferroptotic effects through coordinated regulation of lipid metabolism, iron homeostasis, antioxidant defense, and mTOR-mediated translational control (Figure 9). Future work should focus on delineating the precise molecular pathways through which GBF-8 regulates ferroptosis in radiation-induced brain injury and identifying the specific bioactive constituents responsible for its neuroprotective effects.

## Data Availability

The original contributions presented in this study are included in the article/Supplementary Material. Further inquiries can be directed to the corresponding authors.

## Supplementary Materials

The following supporting information can be downloaded at: https://www.mdpi.com/article/10.3390/antiox15020183/s1, Table S1: Differential lipid metabolites between the GBF-8 and the erastin groups were analyzed by lipidomics; Table S2: Differential protein between the Erastin and the Control groups was analyzed by proteomics. Table S3: Differential protein between the Erastin+GBF-8 and the Erastin groups analyzed by proteomics. Table S4: Analysis and identification of chemical constituents in GBF-8

## Abbreviations

The following abbreviations are used in this manuscript:

Acyl-CoA Synthetase Long-Chain Family Member 4 (Acsl4), ADP Ribosylation Factor 6 (Arf6), Amifostine (Ami), Arachidonate 12-Lipoxygenase (Alox12), BCL2 Binding Component 3 (Bbc3), Bicinchoninic Acid (BCA), B-Rapidly Accelerated Fibrosarcoma (Braf), Calcein-AM/Propidium Iodide (Calcein-AM/PI), C-C Motif Chemokine Ligand 22 (Ccl22), C-C Motif Chemokine Ligand 6 (Ccl6), C-C Motif Chemokine Ligand 9 (Ccl9), CDGSH Iron Sulfur Domain 2 (Cisd2), Chloroform (GBF-2), C-X-C Motif Chemokine Receptor 3 (Cxcr3),Deoxyribonucleic acid (DNA), Doublecortin (DCX), Ethyl acetate (GBF-3), Eukaryotic Translation Initiation Factor 4E Binding Protein 1 (Eif4ebp1), Ferritin Heavy Chain 1 (Fth1), Ferrostatin-1 (Fer-1), Gene Ontology (GO), Ginkgo biloba flower total extract (GBF-1),Glial Fibrillary Acidic Protein (GFAP), Glutamate-Cysteine Ligase Catalytic Subunit (Gclc), Glutaminase 2 (Gls2),Hemoglobin (HGB), Interleukin 10 Receptor Subunit Alpha (Il10ra), Ionizing Radiation (IR), Iron Responsive Element Binding Protein 2 (Ireb2), Kyoto Encyclopedia of Genes and Genomes (KEGG), Lipid peroxidation (LPO), Lysophosphatidylcholines (LysoPCs), Morris Water Maze (MWM), NAD (P)H: Quinone Dehydrogenase 1 (Nqo1), n-butanol (GBF-4), NADPH Oxidase 4 (Nox4), Nuclear Factor, Erythroid 2 Like 2 (Nfe2l2), Novel Object Recognition (NOR), Nuclear Receptor Coactivator 4 (Ncoa4), Open Field Test (OFT), integrated optical density (IOD), Pannexin 2 (Panx2), Parallel reaction monitoring (PRM), Partial least squares-discriminant analysis (PLS-DA), Phosphate-buffered saline (PBS), Phosphatidylcholine (PC), phosphatidylethanolamines (PEs), Platelet (PLT), polyunsaturated fatty acid (PUFA), Principal component analysis (PCA), Reactive Oxygen Species (ROS), Red blood cell (RBC), solute carrier family 1 member 5 (Slc1a5), Solute carrier family 7 member 11 (Slc7a11), Specific pathogen-free (SPF), activator of transcription 3 (Stat3), SUMO2-conjugating enzyme UBC9-interacting protein (Surf2), Transmission electron microscopy (TEM), quadrupole-Orbitrap high-resolution mass spectrometry (UHPLC-Q-Orbitrap HRMS), water (GBF-5), Western Blot (WB), White blood cell (WBC), 25% ethanol (GBF-6), 50% ethanol (GBF-7), and 75% ethanol (GBF-8).
