# Supplementary material for: Active Components of Ginkgo biloba Flower Attenuate Radiation-Induced Cognitive Impairment via Inhibiting Ferroptosis
Source: Antioxidants (Basel). 2026 Feb 1;15(2):183. doi: 10.3390/antiox15020183 (PMC12938514; doi:10.3390/antiox15020183)
Supplement: Supplementary file 1 [file antioxidants-15-00183-s001.zip › antioxidants-4107988-supplementary.pdf]

Supplementary Data for

Active Components of *Ginkgo Biloba* Flower Attenuate Radiation-Induced Cognitive Impairment via Inhibiting Ferroptosis

Ruihong Li[1,2], Yuying Wang[2], Xin Sun[2], Ziming Xia[2], Ying Tian[2], Biqiong Chen[2], Shuchen Liu[2], Min Li[2,*], Xinlong Yan[1,*]

1 Beijing Key Laboratory of Environmental and Viral Oncology, College of Chemistry and Life Science, Beijing University of Technology, Beijing 100124, China; lrh1217021@163.com (R.L.).

2 Beijing Institute of Radiation Medicine, Beijing 100850, China; 1195271281@qq.com (Y.W.); sunx0102@163.com (X.S.); zmxia22@163.com (Z.X.); tianying1977@126.com (Y.T.); chenbiqiong0501@163.com (B.C.) ; liusc118@163.com (S.L.).

* Correspondence: limin82057@163.com (M.L.); yxlong2000@126.com(X.Y.).

2. Materials and methods

2.11 Cell culture and Ferroptosis Assays

Transmission Electron Microscopy

PC12 cells were seeded in 6-well plates and cultured. Following treatment with 20 μM Erastin, cells were washed three times with PBS and fixed with 2.5% glutaraldehyde for 24 h. Subsequently, the samples were post-fixed in 1% osmium tetroxide. After dehydration through a graded ethanol-acetone series, the cells were embedded in epoxy resin, sectioned, stained, and examined by transmission electron microscopy.

Live/Dead Staining

PC12 cells were seeded in 6-well plates. At 85% confluence, the cells were treated with Erastin (10 μM) and various concentrations of GBF-8 (50, 100, and 200 μg/mL). Ferrostatin-1 (Fer-1) served as a positive control for ferroptosis inhibition. After 24 h of incubation, the cells were stained with Calcein-AM and propidium iodide (PI) for 15 minutes and visualized under a fluorescence microscope.

Detection of Intracellular Lipid Peroxidation (LPO) and Ferrous Ions (Fe^{2+})

Intracellular lipid peroxidation (LPO) and ferrous ion levels were assessed using C11-BODIPY 581/591 (5 μM) and FerroOrange (10 μM), respectively. For ferrous ion detection, cells in 6-well plates were stained with FerroOrange for 30 min at 37°C, quantified via flow cytometry. For LPO measurement, cells were incubated with C11-BODIPY 581/591 for 30 min at 37°C and analyzed by flow cytometry according to the manufacturer's protocol.

Intracellular Reactive Oxygen Species (ROS) Detection

PC12 cells were concurrently treated with GBF-8 at concentrations of 50, 100, and 200 μg/mL for 6 h at 37°C, with Fer-1 serving as a positive control for ferroptosis inhibition. Following incubation, the culture medium was removed, and the cells were washed twice with PBS. Subsequently, the cells were incubated with 2 μM DCFH-DA, a fluorescent ROS probe, for 20 min. After staining, the cells were washed three times with PBS to remove any residual extracellular DCFH-DA. Finally, the cells were collected, and the fluorescence intensity was measured by flow cytometry.

Glutathione (GSH) Assay

The ferroptosis model was induced as described above. After 6 h of Erastin treatment, intracellular reduced glutathione (GSH) levels in PC12 cells were quantified using a commercial assay kit according to the manufacturer's instructions.

2.12 Targeted Lipidomics by Mass Spectrometry

Lipid Extraction.

PC12 cells were treated with Erastin for 6 h, harvested by trypsinization and centrifugation, and subjected to lipid extraction. The cell pellets were vortexed for 1 h with 300 μL of methanol and 1 mL of methyl tert-butyl ether (MTBE). Subsequently, 250 μL of water was added, and the mixture was centrifuged at 12,000 rpm and 4 °C for 10 min. A 400 μL aliquot of the upper MTBE phase was collected, dried under nitrogen, and reconstituted in 90 μL of isopropyl

alcohol/acetonitrile (1:1, v/v) containing 3 μL of PC (12:0/13:0) stock solution (15.16 μmol/L), 5 μL of SPH/CER, and 5 μL of an internal standard solution (FFA19:0, 0.25 μg/mL). The resulting mixture was centrifuged again under the same conditions, and the supernatant was transferred to a 250 μL vial insert for subsequent UPLC-MS/MS analysis.

UPLC-MS/MS Method

Targeted lipidomic profiling was performed using a UPLC I-Class system (Waters Corp., Milford, MA, USA). Separation was achieved on a Waters UPLC BEH C8 column (2.1 mm × 100 mm, 1.7 μm) maintained at 40 °C. The mobile phase consisted of (A) acetonitrile/water (60:40, v/v) and (B) isopropyl alcohol/acetonitrile (90:10, v/v), both containing 5 mmol/L ammonium acetate and 0.1% formic acid. The following gradient was applied at a flow rate of 0.26 mL/min: 0–1.5 min, 68% A; 1.5–15.5 min, 68% to 15% A; 15.5–15.6 min, 15% to 3% A; 15.6–18.0 min, 3% A; 18.0–18.1 min, 3% to 68% A; 18.1–20.0 min, 68% A. The injection volume was 3 μL.

Mass spectrometric detection was performed using an electrospray ionization (ESI) source. The source and desolvation temperatures were set to 150 °C and 500 °C, respectively. In positive ion mode, the capillary and cone voltages were 3.0 kV and 30 V, respectively; in negative ion mode, they were -1.8 kV and 29 V. Nitrogen was used as the cone and desolvation gas at flow rates of 150 L/h and 1000 L/h, respectively. Argon was employed as the collision gas at a flow rate of 0.15 mL/min.

2.13 Proteomic Analysis

Protein samples were reduced with 5 mM dithiothreitol (DTT) at 37°C for 1 h and alkylated with 10 mM iodoacetamide (IAA) in the dark at room temperature for 45 min. The mixture was then diluted fourfold with 25 mM ammonium bicarbonate and digested with trypsin (1:50, enzyme-to-protein ratio) overnight at 37°C. Digestion was terminated by acidification with formic acid to a pH < 3.

Peptides were desalted using a C18 solid-phase extraction column pre-activated with 100% acetonitrile and equilibrated with 0.1% formic acid. After sample loading, the column was washed with 0.1% formic acid, and peptides were eluted with 70% acetonitrile. The eluate was collected and lyophilized for subsequent analysis.

UPLC-MS/MS Analysis

PC12 cell samples were subjected to label-free untargeted proteomic analysis using an Orbitrap Exploris™ 480 mass spectrometer with Nanospray Flex™ (NSI) ion source. The injection volume was 1 μg, and the flow rate was 600 nL/min. The mobile phases consisted of eluent A (100% water) and eluent B (80% ACN) with 0.1% formic acid. The gradient program was as follows: 0-5 min, 8-12% B; 5-35 min, 12-30% B; 35-44 min, 30-40% B; 44-45 min, 40-95% B; 45-60 min, 95% B. The data-dependent acquisition mode was used for mass spectrometry, covering the m/z range 350-1500. Full MS scans were acquired over m/z 350-1500 at a resolution of 120,000 (at m/z 200), with an automatic gain control (AGC) target of 300% and a maximum injection time of 50 ms. MS/MS scans were performed in "Top Speed" mode with a resolution of 15,000 (at m/z 200), an AGC target of 75%, a maximum injection time of 22 ms, and a normalized collision energy of 33%. The raw data were saved in .raw format.

Tables:

Table S1. Differential lipid metabolites between the GBF-8 and the erastin groups were analyzed by lipidomics.

No.	Metabolites	VIP Value	Fold change	p Value
1	Cer(d18:3/22:1)	1.10177	0.576785	0.0003153
2	PC(15:0/22:4)	1.14806	1.41261	3.64E-09
3	PC(15:0/22:5)	1.12637	1.61456	4.59E-05
4	PC(16:0/16:0)	1.10272	1.07951	0.0002761
5	PC(16:0/20:5)	1.10086	1.1807	0.0062472
6	PC(16:1/16:1)	1.02269	0.762987	0.0048398

7	PC(16:1/22:6)	1.11931	1.22081	6.40E-05
8	PC(17:0/22:4)	1.1052	1.31367	0.0001498
9	PC(17:0/22:5)	1.08728	1.37587	0.000373
10	PC(17:0/22:6)	1.08972	1.19097	0.0007614
11	PC(17:1/18:3)	1.08535	1.20195	0.000668
12	PC(18:0/20:3)	1.12435	1.27974	3.97E-05
13	PC(18:0/20:4)	1.14888	1.75872	9.42E-09
14	PC(18:1/20:4)	1.12979	1.27268	2.55E-05
15	PC(18:2/18:2)	1.13625	1.42861	8.35E-06
16	PC(20:0/20:5)	1.14329	1.44979	1.03E-06
17	PC(20:4/22:5)	1.00327	1.1366	0.0030897
18	PC(20:5/22:5)	1.05387	1.19515	0.0024701
19	PC(O-16:0/20:5)	1.14029	1.82899	2.00E-06
20	PC(O-16:1/16:1)	1.14156	1.66878	2.20E-06
21	PC(O-18:0/22:5)	1.13855	1.35817	2.51E-06
22	PC(O-18:0/22:4)	1.11128	1.20631	0.0001463
23	PC(O-18:0/20:5)	1.10416	1.24283	0.0001301
24	PC(O-18:1/20:3)	1.14603	1.4715	8.93E-08
25	PC(O-18:1/20:4)	1.14821	1.51808	1.33E-08
26	PC(O-18:1/22:6)	1.1272	1.53416	2.72E-05
27	PC(O-18:2/20:5)	1.13781	1.65682	7.31E-06
28	PC(O-18:2/22:6)	1.13091	1.44082	1.05E-05
29	PC(O-18:2/20:4)	1.13406	2.04998	9.87E-06
30	PC(O-20:1/22:6)	1.08282	1.28576	0.0007791
31	PC(O-20:0/22:5)	1.13606	1.41818	4.24E-06
32	PC(O-22:0/22:4)	1.0199	1.14128	0.0002327
33	TG(14:0/18:2/22:6)	1.13082	1.51065	2.03E-05
34	TG(15:0/18:2/20:5)	1.13681	2.12668	6.07E-06
35	TG(16:0/16:0/20:5)	1.13873	1.83714	5.82E-06
36	TG(16:0/17:1/18:1)	1.13613	1.43892	8.37E-06
37	TG(16:0/18:1/22:6)	1.05974	1.16091	0.0021045

38	TG(16:0/18:2/20:5)	1.13569	1.99238	5.73E-06
39	TG(16:0/18:2/22:6)	1.13379	1.50554	1.34E-05
40	TG(16:1/18:2/20:5)	1.13602	2.68569	7.80E-06
41	TG(17:0/18:1/20:4)	1.09067	1.53558	0.000464
42	TG(18:0/18:1/20:4)	1.04819	1.29765	0.0027771
43	TG(18:1/18:2/22:4)	1.09079	1.28881	0.0009751
44	TG(18:1/18:2/20:5)	1.10604	1.80954	0.0002983
45	TG(18:1/18:2/22:5)	1.12665	1.33226	4.25E-05
46	TG(18:1/18:2/22:6)	1.08836	1.39979	0.0004989
47	TG(18:2/18:2/20:4)	1.08298	1.47646	0.0008542
48	TG(18:4/16:1/18:1)	1.14415	2.6365	4.30E-07
49	TG(24:1/18:2/18:2)	1.03639	0.922276	0.0023179
50	PE(18:0/20:3)	1.10488	1.17895	0.0002595
51	PE(O-16:1/22:6)	1.10046	1.25157	0.0006241
52	PE(O-18:0/22:6)	1.10362	1.27413	0.0005724
53	PE(O-18:3/20:4)	1.11583	1.51807	0.0003285
54	LysoPC(15:0)	1.10981	2.31837	0.0002672
55	LysoPC(16:0)	1.11299	1.62059	0.0001735
56	LysoPC(16:1)	1.10444	3.08963	0.0004599
57	LysoPC(16:2)	1.10324	2.59914	0.0004668
58	LysoPC(17:1)	1.10428	2.98315	0.0004235
59	LysoPC(18:1)	1.09916	2.11073	0.000523
60	LysoPC(19:1)	1.08002	1.57256	0.001217
61	LysoPC(20:3)	1.1034	3.89194	0.000512
62	LysoPC(20:4)	1.1137	4.62293	0.0002328
63	LysoPC(20:5)	1.10004	4.3261	0.0007279
64	LysoPC(22:1)	1.01644	1.28349	0.0039757
65	LysoPC(22:5)	1.10746	3.19244	0.0003312
66	LysoPC(22:6)	1.09817	3.41364	0.0006836
67	LysoPC(O-18:1)	1.10428	2.98315	0.0004235
68	LysoPC(O-20:0)	1.08166	1.45423	0.0007933

69	LysoPE(16:0)	1.1029	1.36885	0.0004488
70	LysoPE(16:1)	1.11114	2.29195	0.0003122
71	LysoPE(17:0)	1.10537	1.50693	0.0003183
72	LysoPE(18:0)	1.11328	1.4575	0.0002087

Table S2. Differential protein between the Erastin and the Control groups was analyzed by proteomics.

Protein	Fold change	pValue	False discovery rate	Sig
Nqo1	1.57697242	0.015735	0.040018	1
Eif4ebp1	3.38404611	0.000741	0.010743	1
Slc1a5	1.42646296	0.001713	0.016558	1
Stat3	0.61871286	0.01407	0.040018	-1
Surf2	1.45169284	0.006816	0.032169	1

Table S3. Differential protein between the Erastin+GBF-8 and the Erastin groups analyzed by proteomics.

Protein	Fold change	pValue	False discovery rate	Sig
Nqo1	0.38850915	0.003034	0.014666	-1
Eif4ebp1	0.31220058	0.002372	0.014666	-1
Slc1a5	0.75408592	0.038009	0.08479	-1
Stat3	1.40413589	0.024467	0.064504	1
Surf2	0.57388547	0.000126	0.001219	-1

Table S4. Analysis and identification of chemical constituents in GBF-8

No.	Retention time (min)	Compound name	Molecular formula	m/z	Fragment ion peak (m/z)	Adducts	Reference
1	0.91	Stachydrine	C7H13NO2	144.1019	144.102, 87.0452	M+H	[1]
2	1.27	nicotinamide	C6H6N2O	123.0557	123.0556, 95.05, 80.0506, 97.0084, 123.0450	M+H	[2]
3	1.5	Dl-isoleucine	C6H13NO2	132.102	86.0975, 69.0712, 72.9386	M+H	[3]
4	1.61	α-arbutin	C12H16O7	317.0879	108.019, 271.0838, 128.0332, 161.044, 109.0273, 74.9884, 136.9893, 71.0113, 113.0222, 101.0221	M-H, M+FA-H	[4]
5	2.34	Hordenine	C10H15NO	166.1226	121.0652, 166.1226, 93.0709	M+H	[5]
6	2.53	4-hydroxybenzoic acid	C7H6O3	156.0654	113.9643, 72.9384, 90.9486	M+NH4	[6]
7	2.68	4-methoxypyridoxine	C9H13NO3	184.0968	152.0706, 134.0601, 184.0968	M+H	[7]
8	3.14	3-(4-hydroxy-3-methoxyphenyl) propane-1, 2-diol	C10H14O4	181.0858	113.9643, 131.9744, 159.9692, 105.0342, 72.9384, 139.9819	M+H-H2O	[6]
9	3.4	Cardiospermin	C11H17NO7	274.0935	274.0936, 89.0219, 115.0378, 71.0114, 59.0113, 100.0381, 112.0381	M-H	[8]
10	4.02	Dl-pantothenic acid	C9H17NO5	220.1177	132.1021, 86.0975, 202.1082, 184.0970, 60.0458, 142.0864, 98.0246, 116.0348, 202.0616, 95.0501	M+H-H2O, M+H, M+Na	[9]

11	4.27	6-hydroxykynurenate	C10H7NO4	206.0446	178.0499, 206.0449	M+H	[10]
12	4.65	Geniposidic acid	C16H22O10	373.1144	373.1145, 167.0698, 211.0603, 193.0493, 165.0541, 134.0356, 59.0114, 72.9905, 122.0346, 89.0217	M-H	[11]
13	5.12	(-)-epigallocatechin -(4*β*→8)-(-)-epicatechin	C30H26O13	593.1311	125.0224, 177.0175, 593.1330, 289.0721, 407.0779, 425.0874, 137.0222, 255.0301, 109.0268, 151.0379	M-H	[12]
14	5.46	3,4-dihydroxybenzaldehyde	C7H6O3	139.0389	116.9663, 111.0447, 80.9462, 137.0597, 93.0344, 139.0393, 65.0399, 97.0657, 98.985, 137.9871	M+H	[13]
15	5.67	Esculin	C15H16O9	339.0723	177.0179, 339.0736	M-H	[6]
16	6.00	Bergenin	C14H16O9	327.0725	147.0431, 192.0053, 327.0724, 193.0127, 207.0333, 59.0113, 312.0493, 165.0539, 234.0162, 91.5731	M-H	[14]
17	6.16	Hypaphorine	C14H18N2O2	247.1437	188.0706, 146.0601, 60.0822, 118.0656, 144.0809, 88.0767	M+H	[15]
18	6.36	Quercetin -3-O-rhamnoside -7-O-(6-feruloylglucosyl -(1→3)-rhamnoside)	C33H40O22	787.1951	315.0151, 287.0200, 478.0744, 787.1997, 625.1391	M-H	
19	6.44	Isorhamnetin -3-arabinoglucoside	C27H30O16	611.1599	287.055, 611.1603, 449.1076	M+H	[13]
20	6.98	Esculetin	C9H6O4	179.0337	179.0338, 123.0445, 133.0286, 151.0391	M+H	[6]
21	7.06	3,4,4a,5,6,7 -hexahydro -1,1,4a-trimethyl-2(1H)-naphthalenone	C13H20O	193.1585	193.1588, 99.0813, 109.1019, 135.117, 175.1482, 161.0596,	M+H	

					133.1013, 95.0865, 105.0706, 119.086		
22	7.3	Quercetin-3-O-*β*-*D*-glucosyl-(1-2)-*α*-*L*-rhamnoside	C27H30O17	625.1417	299.0198, 462.0812, 625.1414, 271.0252, 301.0356	M-H	[16]
23	7.96	(-)-epicatechin	C15H14O6	335.0777	179.0335, 135.043, 93.0321, 335.0781, 161.0226, 111.0428, 155.0331, 137.0224, 133.0274, 134.0352	M-H, M+FA-H	[17]
24	8.08	Sinapine thiocyanate	C16H24NO5+	310.1645	251.0912, 309.3012, 175.0391, 310.1651, 207.0649, 147.0442, 119.0497, 70.0665, 141.1387, 98.0972	M+H	[18]
25	8.22	Isovanillic acid	C8H8O4	169.0493	169.0498, 146.9614, 128.9511, 151.0391, 107.0862, 109.0654, 81.071, 111.0811, 93.0709, 111.0448	M+H	[19]
26	8.45	Kaempferol-3-O-*β*-*D*-glucopyranosyl-(1→2)-*α*-*L*-rhamnopyranoside	C27H30O15	593.1518	593.1517, 353.0673, 297.0767, 383.0775, 473.1098, 325.0722, 503.1177, 296.0681, 295.0615, 365.0666	M-H	[20]
27	9.34	Neoisorutin	C27H30O16	611.1598	633.1425, 471.0898, 308.0297	M+H, M+Na, 2M+H	
28	9.44	Isorhamnetin -3-O-rutinoside	C28H32O16	623.1622	315.0518, 313.0362, 623.1615, 300.0274, 270.017, 476.0961, 316.0222, 242.0219, 229.0285, 298.0126	M-H	[6]

29	9.46	Pinoresinol diglucoside	$C_{32}H_{42}O_{16}$	727.2467	519.1883, 89.0219, 59.0113, 71.0113, 681.2406, 119.0327, 101.0219, 357.135, 179.055, 113.0222	M-H, M+FA-H	[6]
30	9.47	Myricetin	$C_{15}H_{10}O_8$	319.0442	319.0449, 153.0183, 245.0444, 217.0497	M+H	[21]
31	9.96	Eucarvone	$C_{10}H_{14}O$	151.1116	151.1119, 93.0708, 109.0655, 107.0862, 95.0501, 81.071, 151.0755, 109.1019, 91.0552, 95.0864	M+H	[20]
32	10.35	Typhaneoside	$C_{34}H_{42}O_{20}$	769.2207	314.0442, 299.0211, 285.0398, 769.2238, 618.3473, 271.0263, 255.0314, 713.0514, 756.5248, 94.3293	M-H, M+FA-H	[20]
33	10.41	Bilobalide	$C_{15}H_{18}O_8$	325.0928	163.1111, 193.1196, 325.0955, 251.092	M-H	[6]
34	10.48	Rutin	$C_{27}H_{30}O_{16}$	609.1464	300.0278, 609.1481, 271.0252, 255.0300, 151.0016	M-H	[6]
35	10.55	Quercetin -3-O-rhamnopyranoside	$C_{21}H_{20}O_{11}$	447.0934	447.0940, 300.0277, 271.0252, 255.0297, 243.0294, 227.0343	M-H	[6]
36	10.55	Quercetin 3 -O-*α*-*L*-[6'''-p-coumaroyl -*β*-*D*-glucopyranosyl -(1→2)-rhamnopyranoside] -7-O-*β*-*D*-glucopyranoside	$C_{42}H_{46}O_{23}$	917.2373	609.1476, 299.0198, 271.0252, 300.0277, 917.2374, 462.0813, 255.0299, 87.0062, 151.0018, 755.1839	M-H	[6]
37	10.69	Kaempferol 3 -O-*α*-*L*-rhamnopyranosyl(1 →6)-*β*-*D*-galactopyranoside	$C_{27}H_{30}O_{15}$	595.1647	287.0550, 85.0295, 449.1076, 71.0504	M+H, M+Na, 2M+H	[6]

38	10.7	Kaempferol 3 -O-rhamnoside	C21H20O10	431.0982	285.0404, 431.0988, 255.0298, 284.0330, 227.0344, 229.0502	M-H	[20]
39	10.74	Kaempferol-3 -O-*α*- *L* -[6'''-p-coumaroyl -*β*- *D* -glucopyranosyl -(1→2)-rhamnopyranoside] -7-O-*β*- *D* -glucopyranoside	C42H46O22	901.2424	284.0327, 255.0298, 593.1523, 739.1896, 901.2433, 227.0343, 283.0249, 145.0275	M-H	[6]
40	10.82	Hyperoside	C21H20O12	465.102	487.0844, 325.0305, 185.0422, 70.0666, 324.0218	M+H, M+Na	[22]
41	10.96	Ginkgolide J	C20H24O10	469.1351	423.1302, 367.1397	M-H, M+FA-H	[23]
42	11.01	Laricitrin	C16H12O8	333.0596	333.0603, 318.0369, 153.0181, 301.0342	M+H	[24]
43	11.23	Ginkgolide C	C20H24O11	439.1247	59.0114, 125.0223, 323.0991, 71.0113, 439.1249, 383.136, 113.0218, 89.0218, 148.0511, 101.0218	M-H, M+FA-H	[6]
44	11.25	Gibberellic acid	C19H22O6	347.1481	137.0598, 285.1119, 311.1276, 221.0599, 253.0858, 267.1015, 279.1017, 299.1279, 225.0902, 151.0756	M+H-H2O, M+H	[11]
45	11.29	Isoquercitrin	C21H20O12	463.0887	301.0347, 463.0889, 300.0277, 272.0326	M-H	[6]
46	11.45	Tricetin	C15H10O7	303.0492	303.0497, 153.0183, 229.0493	M+H	[25]
47	11.45	Quercetin	C15H10O7	303.4092	303.0499, 304.0532, 153.0184, 229.0497, 137.0236, 257.0447	M+H	[6]
48	11.58	Cynaroside	C21H20O11	449.1069	287.055, 85.0295	M+H	[26]

49	11.58	Nicotiflorin	C27H30O15	593.1512	285.0403, 593.1508, 284.0328, 255.0297, 227.0341, 229.0502	M-H	[6]
50	11.69	Cosmetin	C21H20O10	431.0985	269.0457, 431.0986, 151.0018, 225.0552	M-H	[6]
51	11.71	Perlolyrine	C16H12N2O2	265.0966	265.0974, 206.0839, 247.0873, 219.0928, 207.0896	M+H	[24]
52	11.88	(e)-4-(4-hydroxy-2,6,6-trimethylcyclohexen -1-yl) but-3-en-2-one	C13H20O2	209.1531	209.1524, 165.1267, 109.0654, 133.1013, 175.148, 149.0962, 119.086, 107.0862, 95.0865, 121.1015	M+H	
53	11.92	Isorhamnetin-3-O-neohespeidoside	C28H32O16	625.1749	317.0656, 625.1762, 302.0421, 479.1184	M+H, M+Na, 2M+H	[6]
54	11.92	Tectoridin	C22H22O11	463.1224	317.0651, 71.0504, 85.0297, 302.0415, 301.0704, 129.0548	M+H	[27]
55	11.94	Astragalin	C21H20O11	449.1069	471.0902, 309.0365, 469.2044	M+H, M+Na	[6]
56	12.09	Syringetin -3-rutinoside	C29H34O17	653.173	345.0611, 653.1776, 329.0299, 344.0539, 315.0154, 286.0121, 270.0175, 258.0166, 301.0359, 242.0217	M-H, M+FA-H	
57	12.29	Sophoricoside	C21H20O10	433.1121	271.0598, 433.1127, 703.0378	M+H, 2M+H	[28]
58	12.31	Isorhamnetin-3 -galactoside	C22H22O12	477.1038	477.1039, 314.0437, 271.0247, 243.0285, 285.0433, 330.0386, 257.0455, 300.027, 299.0197, 270.0164	M-H	[29]
59	12.53	Kaempferol -3-arabofuranoside#	C20H18O10	417.083	284.0329, 417.0836, 255.0298, 227.0344	M-H	[28]

60	12.60	Hesperidin	C28H34O15	609.183	301.0719, 609.185, 164.0098, 151.0018, 286.0485	M-H	[30]
61	12.81	Thermopsoside	C22H22O11	463.1226	301.0706, 286.0471, 463.1237, 258.0521	M+H, 2M+H	[31]
62	12.89	Trifolin	C21H20O11	449.107	287.055, 449.1101	M+H	[32]
63	13.09	Rhoifolin	C27H30O14	577.1571	269.0457, 577.1563, 211.0393, 239.0346, 268.0381, 213.0551, 241.0505	M-H	[33]
64	13.34	Quercetin-3 -O-*α*-*L*-[6'''-p-coumaroyl -*β*-*D*-glucopyranosyl -(1→2)-rhamnopyranoside]	C36H36O18	755.1834	300.0277, 271.0252, 755.1854, 255.0298, 609.1447, 243.0299, 151.0018	M-H	[6]
65	13.65	coniferin	C16H22O8	387.1302	193.0492, 178.0260, 387.1078, 134.0353, 194.0531, 149.0585	M+FA-H	[34]
66	13.76	Bilobanone	C15H20O2	215.1427	215.1432, 95.0864, 105.0706, 81.0711, 131.0857, 145.1014, 107.0863, 93.0709, 157.1014, 109.1018	M+H-2H2O	[35]
67	13.76	Baicalin	C21H18O11	445.0781	269.0458, 443.1913, 59.0113, 445.0768, 113.0221, 71.0113, 85.027, 237.1497	M-H	[36]
68	14.17	Daidzein	C15H10O4	255.0644	255.065, 199.0754, 137.0233, 57.0714	M+H	[28]
69	14.32	Kaempferol-3 -O-*α*-*L* - [6'''-p-coumaroyl -*β*-*D*-glucopyranosyl -(1→2)-rhamnopyranoside]	C36H36O17	739.1887	284.0329, 255.0297, 739.1903, 227.0345, 593.153, 145.0273	M-H	[6]

70	14.41	Isorhamnetin-3 - O-*α*-*L* - [6'-p-coumaroyl -*β*-*D* - glucopyranosyl -(1→2)-rhamnopyranoside]	C37H38O18	769.1993	284.033, 255.0298, 227.0343, 769.2026, 593.1494	M-H, M+FA-H	[28]
71	14.59	Ginkgolide A	C20H24O9	409.1484	373.1282, 355.1181, 95.0865, 81.0711, 313.0695, 253.0863, 93.0709, 137.06, 105.0707, 69.0712	M+H-2H2O	[6]
72	14.63	Ginkgolide B	C20H24O10	423.1299	61.9858, 421.2068, 59.0113, 423.1311, 367.1397, 125.0221, 89.0218, 113.0221, 101.0221	M-H, M+FA-H	[6]
73	15.01	Luteolin	C15H10O6	285.0405	285.0406, 133.0273, 151.0016	M-H	[6]
74	15.28	Andrographolide	C20H30O5	349.2023	349.2053, 331.1927, 348.1818, 139.0743, 102.0536, 58.0036, 59.0113	M-H	[37]
75	15.42	Tiliroside	C30H26O13	593.131	284.0331, 285.0406, 255.0304, 593.1299, 227.0346, 145.0267, 285.077, 593.1377, 151.0013	M-H	[38]
76	15.67	Calycosin	C16H12O5	283.0612	283.0614, 281.1355, 268.0382, 237.1495, 281.1033, 191.1066, 219.102, 189.1273, 240.0424	M-H	[39]
77	16.06	Wogonoside	C22H20O11	461.1070	285.0757, 270.0522, 461.1082	M+H	[40]
78	16.32	trans-caffeic acid	C9H8O4	163.0386	163.0390, 135.0442, 133.0287,95.05	M+H-H2O	[28]
79	16.5	Naringenin	C15H12O5	271.0611	271.0616, 151.0018, 119.048, 107.0115, 65.0007, 93.0321, 177.0179, 83.0113	M-H	[41]
80	16.72	Silibinin	C25H22O10	481.1142	481.1149, 125.0223, 152.0097, 151.0013, 178.9976, 301.035,	M-H	[42]

					78.9567, 124.0146, 273.0409, 119.0478		
81	16.76	Genistein	C15H10O5	271.0593	271.0599, 153.0183	M+H	[39]
82	16.93	Hesperetin	C16H14O6	301.0715	301.0716, 151.0018, 134.0352, 149.0589, 107.0115, 65.0007, 164.0101	M-H	[43]
83	16.94	4, 4′-dihydroxy-3, 3′-imino-di-benzoic acid	C14H11NO6	289.0594	287.055, 288.0583, 153.0184, 121.0289	M+H	[27]
84	16.97	Kaempferol	C15H10O6	285.0403	285.0407	M-H	[6]
85	17.2	Diosmetin	C16H12O6	301.0699	301.0707, 286.0472, 258.0522	M+H	[44]
86	17.36	Isorhamnetin	C16H12O7	317.0648	317.0656, 318.0690, 153.0184, 302.0422, 274.0472	M+H	[6]
87	17.53	(1ar,4as,7r,7ar,7br) -1,1,7-trimethyl -4-methylidenedecahydro -1H-cyclopropa(e)azulen -7-ol	C15H24O	221.1895	81.071, 107.0862, 93.0708, 105.0706, 119.086, 219.1744, 135.117, 95.0865, 221.1898, 133.1014	M+H	[28]
88	17.71	Syringetin	C17H14O8	347.0754	347.076, 331.0444, 258.0523, 332.0511, 153.0183, 303.0497	M+H	[43]
89	17.75	Tricin	C17H14O7	331.0803	331.0811, 315.0498, 316.0555, 270.0522, 86.0612, 287.0544	M+H	[39]
90	18.03	Amentoflavone	C30H18O10	537.0827	537.0833, 375.0515, 331.0615, 417.0618, 443.0416, 307.0614, 399.051, 117.0322, 333.0402	M-H	[6]
91	18.34	Apigenin	C15H10O5	541.1121	541.1133, 389.0019, 153.0183, 542.1169, 390.1054, 121.0288	2M+H	[45]

92	18.34	Chrysoeriol	$C_{16}H_{12}O_6$	301.0698	301.0707, 258.0522, 91.0551, 286.0471	M+H	[46]
93	18.39	Aurantio-obtusin	$C_{17}H_{14}O_7$	331.0803	331.0813, 316.0576, 288.0627, 228.1019, 81.071, 105.0706, 93.0708, 137.0599, 107.086, 91.0552	M+H	[47]
94	18.77	Ginkgetin 7'' -O-*β*-*D*-glucopyranoside	$C_{38}H_{32}O_{15}$	727.1684	565.1147, 533.0891, 727.1672, 374.0432, 389.0671, 151.0014, 517.057, 107.0115, 401.0299	M-H, M+FA-H	[48]
95	18.93	Isoginkgetin -7-O-*β*-*D*-glucopyranoside	$C_{38}H_{32}O_{15}$	729.1795	567.1291, 135.0443, 729.1811, 535.1022	M+H	[48]
96	18.93	3- [5,7-dihydroxy -2-(4-methoxyphenyl)-4-oxo-4H-chromen -8-yl]-4-methoxybenzoic acid	$C_{24}H_{18}O_8$	435.1065	435.1081, 135.0443, 403.0812, 390.0722	M+H	
97	19.02	hexadecanoic acid	$C_{16}H_{32}O_2$	301.2385	240.2321, 301.2849, 241.2355, 57.0714, 95.0865, 71.0868	M+FA-H	[49]
98	19.04	5'-methoxybilobetin	$C_{32}H_{22}O_{11}$	583.1221	583.1238, 121.0288, 551.0983	M+H	[17]
99	19.08	Parthenolide	$C_{15}H_{20}O_3$	249.1478	151.039, 249.1483, 161.0596, 107.0498, 179.0702, 133.0649, 55.0558, 147.0805, 231.1377, 105.0705	M+H	[50]
100	19.15	Chrysin	$C_{15}H_{10}O_4$	255.0644	255.0651	M+H	[51]
101	19.32	Pinocembrin	$C_{15}H_{12}O_4$	255.0660	255.0662, 151.0019, 107.0114, 213.0547	M-H	[52]
102	19.36	Genkwanin	$C_{16}H_{12}O_5$	283.0612	283.0615, 268.0378	M-H	[6]
103	19.37	Casticin	$C_{19}H_{18}O_8$	375.1065	375.1078, 345.0605, 335.1826, 129.0548, 145.065, 215.0187,	M+H	[53]

					93.0708, 197.0083, 327.0504, 95.0864		
104	19.37	Acacetin	C16H12O5	285.0748	285.0757, 242.0572, 270.0519, 167.0339	M+H	[54]
105	19.41	Galangin	C15H10O5	269.0455	269.0458	M-H	[55]
106	19.45	Bilobetin	C31H20O10	551.0988	551.0984	M-H	[6]
107	19.47	Kaempferide	C16H12O6	301.0697	551.1007, 389.0672, 374.041, 375.0516, 431.075, 225.0551, 330.0534, 401.1034, 117.0325, 345.077	M+H	[56]
108	19.52	Nobiletin	C21H22O8	403.1378	301.0708, 258.0528	M+H	[51]
109	19.67	Pectolinarigenin	C17H14O6	315.0853	315.0862, 300.0629, 272.0681, 60.0459, 228.1016	M+H	[41]
110	20.44	Isoginkgetin	C32H22O10	567.127	567.1288, 135.0442, 535.102	M+H, 2M+H	[6]
111	20.98	6-(10Z-Heptadecenyl)salicylic acid	C24H38O3	419.2804	419.2854, 343.2643, 420.2891, 375.2538, 387.2538,231.1741, 344.2673. 121.0274, 111.0065	M+FA-H	[57]
112	21.00	p-coumaric acid	C9H8O3	164.0469	164.1070, 139.9822, 142.9671, 163.0389, 107.0499, 107.0737	M+H-H2O, M+H	[58]
113	21.25	Vitexilactone	C22H34O5	377.2334	377.2336, 378.2371, 333.2439, 245.1909, 247.1702, 217.1585	M-H	[59]
114	21.89	Ruvoside deglycosylation	C23H34O5	389.2336	345.2439, 389.2338, 342.2653, 122.0353, 327.2342, 147.0431, 313.2376	M-H	[6]
115	22.05	Sciadopitysin	C33H24O10	581.1429	581.144, 135.0442, 549.1177	M+H, 2M+H	[6]

116	22.69	6-tridecylresorcylic acid	C20H32O4	319.2258	81.071, 95.0864, 67.0556, 69.0712, 55.0558, 57.0714, 93.0708, 83.0866, 109.1018, 79.0554	M+H-H2O, M+H	[60]
117	22.90	Hydroginkgolinic acid	C21H34O3	333.2437	333.2437, 235.1319, 122.0349, 315.234, 291.2325, 245.1545	M-H	[60]
118	23.92	Bilobol	C21H34O2	317.2485	317.2488, 299.238, 121.0272, 203.1431, 316.2381	M-H	[61]
119	23.98	Fritillebic acid	C22H34O4	361.2385	361.2388, 299.238, 362.2426, 317.2485, 20.51383, 203.1428, 119.0476	M-H	
120	24.08	Scillaren a deglycosylation	C24H32O4	385.2363	367.2264, 81.0711, 383.3307, 95.0865, 55.0194, 67.0557, 69.0712, 55.0558, 93.0707, 107.0498	M+H	[17]
121	24.45	Anacardic acid C	C22H32O3	389.2337	299.238, 343.2278, 119.0479, 121.0273	M+FA-H	[62]
122	25.18	Anacardic acid D	C22H30O3	341.2121	341.2108, 340.2035, 297.2213, 183.0105, 119.0475, 121.0273	M-H	[60]
123	26.42	6-tridecylsalicylic acid	C20H32O3	319.2277	275.2379, 319.2281, 121.0272	M-H	[63]
124	26.46	6-[(8z)-pentadecenyl] -salicylic acid	C22H34O3	347.2577	151.0391, 347.258, 329.2476, 107.0497, 179.0705, 161.0598, 133.0651	M+H-H2O, M+H	[56]
125	27.59	Anacardic acid	C22H36O3	347.2593	341.2108, 340.2035, 297.2213, 183.0105, 119.0475, 121.0273	M-H	[62]

Reference

1. Zhang, Y.; Zhu, S.; Gu, Y.; Feng, Y.; Gao, B. Network Pharmacology Combined with Experimental Validation to Investigate the Mechanism of the Anti-Hyperuricemia Action of Portulaca Oleracea Extract. *Nutrients* **2024**, *16*, 3549, doi:10.3390/nu16203549.
2. ShangGuan, X. Application of Spectrophotometry in Drug Analysis - A Study on the Analytical Methods of Ginkgo Biloba Flavonoids and Nicotinamide. Master, Northwest University, 2004.
3. Sawada, Y.; Akiyama, K.; Sakata, A.; Kuwahara, A.; Otsuki, H.; Sakurai, T.; Saito, K.; Hirai, M.Y. Widely Targeted Metabolomics Based on Large-Scale MS/MS Data for Elucidating Metabolite Accumulation Patterns in Plants. *Plant Cell Physiol.* **2008**, *50*, 37, doi:10.1093/pcp/pcn183.
4. Repert, S.; Matthes, S.; Rozhon, W. Quantification of Arbutin in Cosmetics, Drugs and Food Supplements by Hydrophilic-Interaction Chromatography. *Molecules* **2022**, *27*, 5673, doi:10.3390/molecules27175673.
5. Roman, M.C.; Betz, J.M.; Hildreth, J. Determination of Synephrine in Bitter Orange Raw Materials, Extracts, and Dietary Supplements by Liquid Chromatography with Ultraviolet Detection: Single-Laboratory Validation. *J. AOAC Int.* **2007**, *90*, 68.
6. Li, M. Chemical Constituents from the Male Flowers of Ginkgo Biloba L. and Their Biological Activities. phdthesis, BeiJing Institute of Radiation Medicine: BeiJing, China, 2019.
7. K, W.; S, I.; K, U.; M, S.; M, H. An Antivitamin B6, 4′-Methoxypyridoxine, from the Seed of Ginkgo Biloba L. *Chem. Pharm. Bull. (Tokyo)* **1985**, *33*, 3555–3557, doi:10.1248/CPB.33.3555.
8. Kumar, R.; Murugananthan, G.; Nandakumar, K.; Talwar, S. Isolation of Anxiolytic Principle from Ethanolic Root Extract of Cardiospermum Halicacabum. *Phytomedicine Int. J. Phytother. Phytopharm.* **2011**, *18*, 219–223, doi:10.1016/j.phymed.2010.07.002.
9. Yu, J.; Liu, H.; Xiong, J.; Qu, S.; Xie, X.; Zhao, H.; Zhu, Z.; Wang, Y.; Han, Y. Non-Target Metabolomics Unravels the Effect and Mechanism of Lianpu Drink on Spleen-Stomach Damp-Heat Syndrome. *J. Chromatogr. B Analyt. Technol. Biomed. Life. Sci.* **2024**, *1246*, 124281, doi:10.1016/j.jchromb.2024.124281.
10. Cao, J.; Chen, L.; Li, M.; Cao, F.; Zhao, L.; Su, E. Efficient Extraction of Proanthocyanidin from Ginkgo Biloba Leaves Employing Rationally Designed Deep Eutectic Solvent-Water Mixture and Evaluation of the Antioxidant Activity. *J Pharm Biomed Anal* **2018**, *158*, 317–326, doi:10.1016/j.jpba.2018.06.007.
11. Mfotie Njoya, E.; McGaw, L.J.; Makhafola, T.J. Investigating the Phytochemical Composition, Antioxidant, and Anti-Inflammatory Potentials of Cassinopsis Ilicifolia (Hochst.) Kuntze Extract against Some Oxidative Stress and Inflammation Molecular Markers. *Curr. Issues Mol. Biol.* **2024**, *46*, 9639–9658, doi:10.3390/cimb46090573.
12. Tawaha, K.; Sadi, R.; Qa'dan, F.; Matalka, K.Z.; Nahrstedt, A. A Bioactive Prodelphinidin from Mangifera Indica Leaf Extract. *Z. Naturforschung C J. Biosci.* **2010**, *65*, 322–326, doi:10.1515/znc-2010-5-603.
13. Dou, M.; Huang, J.; Yu, M.; Li, H.; Song, Y.; Peng, Z.; Du, S.; Bai, J. HPLC Combined

with Chemometrics for Quality Control of Huamoyan Granules or Capsules. *Chin. Herb. Med.* **2024**, *16*, 449–456, doi:10.1016/j.chmed.2023.03.005.
14. Js, K.; J, L.; Y, X.; Y, C.; C, Y.; X, Z.; R, Z.; M, A.; W, X.; H, L.; et al. Constituents from Leaves of Macaranga Hemsleyana. *Chin. Herb. Med.* **2023**, *16*, doi:10.1016/j.chmed.2023.03.006.
15. He, Y.; Wu, F.; Tan, Z.; Zhang, M.; Li, T.; Zhang, A.; Miao, J.; Ou, M.; Long, L.; Sun, H.; et al. Quality Markers' Discovery and Quality Evaluation of Jigucao Capsule Using UPLC-MS/MS Method. *Mol. Basel Switz.* **2023**, *28*, 2494, doi:10.3390/molecules28062494.
16. Chen, X.; Kong, L.; Sheng, L.; Li, X.; Zou, H. Applications of Two-Dimensional Liquid Chromatography Coupled to Mass Spectrometry for the Separation and Identification of Compounds in Ginkgo Biloba Extracts. *Chin. J. Chromatogr.* **2005**, *23*, 46–51.
17. Biernacka, P.; Felisiak, K.; Adamska, I. The Potential of Dried Ginkgo Biloba Leaves as a Novel Ingredient in Fermented Beverages of Enhanced Flavour and Antioxidant Properties. *Food Chem.* **2024**, *461*, 141018, doi:10.1016/j.foodchem.2024.141018.
18. Gao, Y.; Xu, M.; Zheng, Z.; Wan, Y.; Wu, S.; Li, C. *Foods* **2022**, *11*, 2014, doi:10.3390/foods11142014.
19. Ellnain-Wojtaszek, M.; Zgórka, G. HIGH-PERFORMANCE LIQUID CHROMATOGRAPHY AND THIN-LAYER CHROMATOGRAPHY OF PHENOLIC ACIDS FROM GINKGO BILOBA L. LEAVES COLLECTED WITHIN VEGETATIVE PERIOD. *J. Liq. Chromatogr. Relat. Technol.* **1999**, doi:10.1081/JLC-100101744.
20. Wu, J.; Huang, H.; Gong, L.; Tian, X.; Peng, Z.; Zhu, Y.; Wang, W. A Flavonoid Glycoside Compound from Siraitia Grosvenorii with Anti-Inflammatory and Hepatoprotective Effects In Vitro. *Biomolecules* **2024**, *14*, 450, doi:10.3390/biom14040450.
21. Baker, T.R.; Regg, B.T. A Multi-Detector Chromatographic Approach for Characterization and Quantitation of Botanical Constituents to Enable in Silico Safety Assessments. *Anal. Bioanal. Chem.* **2018**, *410*, 5143, doi:10.1007/s00216-018-1163-y.
22. Shirai, M.; Kawai, Y.; Yamanishi, R.; Terao, J. Approach to Novel Functional Foods for Stress Control 5. Antioxidant Activity Profiles of Antidepressant Herbs and Their Active Components. *J. Med. Investig. JMI* **2005**, *52 Suppl*, 249–251, doi:10.2152/jmi.52.249.
23. Zhang, F.; Fei, Q.; Huang, X.; Yu, S.; Qiu, R.; Guan, L.; Wu, B.; Shan, M. LC-MS Based Strategy for Chemical Profiling and Quantification of Dispensing Granules of Ginkgo Biloba Seeds. *Heliyon* **2024**, *10*, e36909, doi:10.1016/j.heliyon.2024.e36909.
24. Sun, Y.; Wang, L.; Du, L.; Yu, H.; Tian, Y.; Jin, H.; Li, S.; Yan, S.; Xiao, X. Investigation on the Mechanism of Ginkgo Folium in the Treatment of Non-Alcoholic Fatty Liver Disease by Strategy of Network Pharmacology and Molecular Docking. *Technol. Health Care Off. J. Eur. Soc. Eng. Med.* **2023**, *31*, 209–221, doi:10.3233/THC-236018.
25. Lee, D.; Yu, J.S.; Huang, P.; Qader, M.; Manavalan, A.; Wu, X.; Kim, J.-C.; Pang, C.; Cao, S.; Kang, K.S.; et al. Identification of Anti-Inflammatory Compounds from Hawaiian Noni (Morinda Citrifolia L.) Fruit Juice. *Mol. Basel Switz.* **2020**, *25*, 4968, doi:10.3390/molecules25214968.

26. Zhou, K.; Xiao, S.; Cao, S.; Zhao, C.; Zhang, M.; Fu, Y. Improvement of Glucolipid Metabolism and Oxidative Stress via Modulating PI3K/Akt Pathway in Insulin Resistance HepG2 Cells by Chickpea Flavonoids. *Food Chem. X* **2024**, *23*, 101630, doi:10.1016/j.fochx.2024.101630.
27. Alruwad, M.I.; Salah El Dine, R.; Gendy, A.M.; Saleh, A.M.; Khalaf, M.A.; El Hefnawy, H.M.; Sabry, M.M. Insights into Clematis Cirrhosa L. Ethanol Extract: Cytotoxic Effects, LC-ESI-QTOF-MS/MS Chemical Profiling, Molecular Docking, and Acute Toxicity Study. *Pharm. Basel Switz.* **2024**, *17*, 1347, doi:10.3390/ph17101347.
28. Bampali, E.; Germer, S.; Bauer, R.; Kulić, Ž. HPLC-UV/HRMS Methods for the Unambiguous Detection of Adulterations of Ginkgo Biloba Leaves with Sophora Japonica Fruits on an Extract Level. *Pharm. Biol.* **2021**, *59*, 436, doi:10.1080/13880209.2021.1910717.
29. Jiao, H.; Guan, Q.; Dong, R.; Ran, K.; Wang, H.; Dong, X.; Wei, S. Metabolomics Analysis of Phenolic Composition and Content in Five Pear Cultivars Leaves. *Plants Basel Switz.* **2024**, *13*, 2513, doi:10.3390/plants13172513.
30. Penna-Coutinho, J.; Aguiar, A.C.; Krettli/, A.U. Commercial Drugs Containing Flavonoids Are Active in Mice with Malaria and in Vitro against Chloroquine-Resistant Plasmodium Falciparum. *Mem. Inst. Oswaldo Cruz* **2018**, *113*, e180279, doi:10.1590/0074-02760180279.
31. Hasler, A.; Gross, G.A.; Meier, B.; Sticher, O. Complex Flavonol Glycosides from the Leaves of Ginkgo Biloba. *Phytochemistry* **1992**, *31*, 1391–1394, doi:10.1016/0031-9422(92)80298-s.
32. Ma, B.; Wang, S.; Li, H.; Wang, Q.; Hong, Y.; Bao, Y.-M.; Liu, H.; Li, M.; Zhao, Y.; Guo, L.-P. Combining Metabolomics and Transcriptomics to Reveal the Potential Medicinal Value of Rare Species Glycyrrhiza Squamulose. *Heliyon* **2024**, *10*, e30868, doi:10.1016/j.heliyon.2024.e30868.
33. Zhang, X.; Yu, X.; Sun, X.; Meng, X.; Fan, J.; Zhang, F.; Zhang, Y. Comparative Study on Chemical Constituents of Different Medicinal Parts of Lonicera Japonica Thunb. Based on LC-MS Combined with Multivariate Statistical Analysis. *Heliyon* **2024**, *10*, e31722, doi:10.1016/j.heliyon.2024.e31722.
34. Aoki, D.; Hanaya, Y.; Akita, T.; Matsushita, Y.; Yoshida, M.; Kuroda, K.; Yagami, S.; Takama, R.; Fukushima, K. Distribution of Coniferin in Freeze-Fixed Stem of Ginkgo Biloba L. by Cryo-TOF-SIMS/SEM. *Sci. Rep.* **2016**, *6*, 31525, doi:10.1038/srep31525.
35. Kimura, H.; Irie, H.; Ueda, K.; Ueo, S. The Constituents of the Heartwood of Ginkgo Biloba L. V. The Structure and Absolute Configuration of Bilobanone. *J. Pharm. Soc. Jpn.* **1968**, *88*, 562–572, doi:10.1248/YAKUSHI1947.88.5_562.
36. Yin, J.; Li, C.; Zhang, J.; Ding, H.; Han, L.; Yang, W.; Li, F.; Song, X.; Bie, S.; Yu, H.; et al. Comprehensive Multicomponent Characterization and Quality Assessment of Shuang-Huang-Lian Powder Injection Using Ultra-High-Performance Liquid Chromatography-Quadrupole Time-of-Flight-Mass Spectrometry and Ultra-High-Performance Liquid Chromatography-Quadrupole-Orbitrap-Mass Spectrometry. *Rapid Commun. Mass Spectrom.* **2023**, *37*, e9479, doi:10.1002/rcm.9479.
37. Songvut, P.; Pholphana, N.; Suriyo, T.; Rangkadilok, N.; Panomvana, D.; Puranajoti, P.; Satayavivad, J. A Validated LC-MS/MS Method for Clinical Pharmacokinetics and

Presumptive Phase II Metabolic Pathways Following Oral Administration of Andrographis Paniculata Extract. *Sci. Rep.* **2023**, *13*, 2534, doi:10.1038/s41598-023-28612-1.
38. Lephatsi, M.M.; Choene, M.S.; Kappo, A.P.; Madala, N.E.; Tugizimana, F. An Integrated Molecular Networking and Docking Approach to Characterize the Metabolome of Helichrysum Splendidum and Its Pharmaceutical Potentials. *Metabolites* **2023**, *13*, 1104, doi:10.3390/metabo13101104.
39. Yang, T.; Deng, F.; Yang, X.; Li, S. Establishment of HPLC Fingerprints for Feiqizhong Tablets and Simultaneous Determination of Fourteen Constituents. *J. Anal. Methods Chem.* **2024**, *2024*, 7703951, doi:10.1155/2024/7703951.
40. Wenya Gao; Tao Li; Yanyan Zhou; Mingli Li; Linna Wang Qualitative and Quantitative Study on Chemical Constituents in Scutellariae Radix Decoction. *Chin. Tradit. Herb. Drugs* **2022**, *53*, 7339–7352.
41. Qiu, J.; Chen, X.; Netrusov, A.I.; Zhou, Q.; Guo, D.; Liu, X.; He, H.; Xin, X.; Wang, Y.; Chen, L. Screening and Identifying Antioxidative Components in Ginkgo Biloba Pollen by DPPH-HPLC-PAD Coupled with HPLC-ESI-MS2. *PLOS ONE* **2017**, *12*, e0170141, doi:10.1371/journal.pone.0170141.
42. Muchiri, R.N.; van Breemen, R.B. Chemical Standardization of Milk Thistle (Silybum Marianum L.) Extract Using UHPLC-MS/MS and the Method of Standard Addition. *J. Am. Soc. Mass Spectrom.* **2024**, *35*, 1726–1732, doi:10.1021/jasms.4c00125.
43. Otsuka, H.; Zhong, X.N.; Hirata, E.; Shinzato, T.; Takeda, Y. Myrsinionosides A-E: Megastigmane Glycosides from the Leaves of Myrsine Seguinii Lev. *Chem. Pharm. Bull. (Tokyo)* **2001**, *49*, 1093–1097, doi:10.1248/cpb.49.1093.
44. Ni, J.; Zhang, N.; Zhan, Y.; Ding, K.; Qi, P.; Wang, X.; Ding, W.; Xu, M. Transgenic Tobacco Plant Overexpressing Ginkgo Dihydroflavonol 4-Reductase Gene GbDFR6 Exhibits Multiple Developmental Defects. *Front. Plant Sci.* **2022**, *13*, 1066736, doi:10.3389/fpls.2022.1066736.
45. Karaźniewicz-Łada, M.; Wójtowski, J.A.; Główka, F.; Danków, R.; Pikul, J.; Gryszczyńska, A.; Foksowicz-Flaczyk, J.; Mikołajczak, P.Ł. Application of UPLC-MS/MS Method for Analysis of Apigenin, Apigenin 7-Glucoside and Chlorogenic Acid in Goat Serum. *Chromatographia* **2023**, *86*, 401–411, doi:10.1007/s10337-023-04250-7.
46. Kim, J.A.; Yang, S.Y.; Wamiru, A.; McMahon, J.B.; Le Grice, S.F.J.; Beutler, J.A.; Kim, Y.H. New Monoterpene Glycosides and Phenolic Compounds from Distylium Racemosum and Their Inhibitory Activity against Ribonuclease H. *Bioorg. Med. Chem. Lett.* **2011**, *21*, 2840–2844, doi:10.1016/j.bmcl.2011.03.091.
47. Qin, S.; Xu, Y.; Li, K.; Gong, K.; Peng, J.; Shi, S.; Yan, F.; Cai, W. Identification of Metabolites of Aurantio-Obtusin in Rats Using Ultra-High-Performance Liquid Chromatography-Q-Exactive Orbitrap Mass Spectrometry with Parallel Reaction Monitoring. *J. Anal. Methods Chem.* **2021**, *2021*, 6630604, doi:10.1155/2021/6630604.
48. Hyun, S.K.; Kang, S.S.; Son, K.H.; Chung, H.Y.; Choi, J.S. Biflavone Glucosides from Ginkgo Biloba Yellow Leaves. *Chem. Pharm. Bull. (Tokyo)* **2005**, *53*, 1200–1201, doi:10.1248/cpb.53.1200.
49. Zhang, Q.; Wang, G.; A, J.; Wu, D.; Zhu, L.; Ma, B.; Du, Y. Application of GC/MS-

Based Metabonomic Profiling in Studying the Lipid-Regulating Effects of Ginkgo Biloba Extract on Diet-Induced Hyperlipidemia in Rats. *Acta Pharmacol. Sin.* **2009**, *30*, 1674–1687, doi:10.1038/aps.2009.173.

50. Qazi, S.S.; Lombardo, D.A.; Abou-Zaid, M.M. A Metabolomic and HPLC-MS/MS Analysis of the Foliar Phenolics, Flavonoids and Coumarins of the Fraxinus Species Resistant and Susceptible to Emerald Ash Borer. *Mol. Basel Switz.* **2018**, *23*, 2734, doi:10.3390/molecules23112734.
51. Bouzenad, N.; Ammouchi, N.; Chaib, N.; Messaoudi, M.; Bousabaa, W.; Bensouici, C.; Sawicka, B.; Atanassova, M.; Ahmad, S.F.; Zahnit, W. Exploring Bioactive Components and Assessing Antioxidant and Antibacterial Activities in Five Seaweed Extracts from the Northeastern Coast of Algeria. *Mar. Drugs* **2024**, *22*, 273, doi:10.3390/md22060273.
52. Yan, B.; Cao, G.; Sun, T.; Zhao, X.; Hu, X.; Yan, J.; Peng, Y.; Shi, A.; Li, Y.; Xue, W.; et al. Determination of Pinocembrin in Human Plasma by Solid-phase Extraction and LC/MS/MS: Application to Pharmacokinetic Studies. *Biomed. Chromatogr.* **2014**, *28*, 1601–1606, doi:10.1002/bmc.3186.
53. Alves, O.J.A.; Ozelin, S.D.; Magalhães, L.F.; Candido, A.C.B.B.; Gimenez, V.M.M.; Silva, M.L.A.E.; Cunha, W.R.; Januário, A.H.; Tavares, D.C.; Magalhães, L.G.; et al. HPLC Method for Quantifying Verbascoside in Stizophyllum Perforatum and Assessment of Verbascoside Acute Toxicity and Antileishmanial Activity. *Front. Plant Sci.* **2023**, *14*, 1324680, doi:10.3389/fpls.2023.1324680.
54. Huang, Y.; Tao, M.; Li, R.; Liang, F.; Xu, T.; Zhong, Q.; Yuan, Y.; Wu, T.; Pan, S.; Xu, X. Identification of Key Phenolic Compounds for Alleviating Gouty Inflammation in Edible Chrysanthemums Based on Spectrum-Effect Relationship Analyses. *Food Chem. X* **2023**, *20*, 100897, doi:10.1016/j.fochx.2023.100897.
55. Xu, X.; Li, X.; Chen, S.; Liang, Y.; Zhang, C.; Huang, Y. Simultaneous Qualitative and Quantitative Analyses of 41 Constituents in Uvaria Macrophylla Leaves Screen Antioxidant Quality-Markers Using Database-Affinity Ultra-High-Performance Liquid Chromatography with Quadrupole Orbitrap Tandem Mass Spectrometry. *Mol. Basel Switz.* **2024**, *29*, 4886, doi:10.3390/molecules29204886.
56. Alqahtani, J.; Negm, W.A.; Elekhnawy, E.; Hussein, I.A.; Hassan, H.S.; Alanzi, A.R.; Moglad, E.; Ahmed, R.; Ibrahim, S.; El-Sherbeni, S.A. Potential Surviving Effect of Cleome Droserifolia Extract against Systemic Staphylococcus Aureus Infection: Investigation of the Chemical Content of the Plant. *Antibiot. Basel Switz.* **2024**, *13*, 450, doi:10.3390/antibiotics13050450.
57. Mengying Chu Improvement of the Extraction Method of Ginkgolic Acid Monomers and Study on Their Antioxidant Activity and Toxicity Evaluation. Master's Thesis, YangZhou University, 2020.
58. Karthikeyan, R.; Devadasu, C.; Srinivasa Babu, P. Isolation, Characterization, and RP-HPLC Estimation of P-Coumaric Acid from Methanolic Extract of Durva Grass (Cynodon Dactylon Linn.) (Pers.). *Int. J. Anal. Chem.* **2015**, *2015*, 201386, doi:10.1155/2015/201386.
59. Ono, M.; Yamamoto, M.; Yanaka, T.; Ito, Y.; Nohara, T. Ten New Labdane-Type Diterpenes from the Fruit of Vitex Rotundifolia. *Chem. Pharm. Bull. (Tokyo)* **2001**, *49*,

82–86, doi:10.1248/cpb.49.82.
60. Wei, J.; Xu, R.; Zhang, Y.; Zhao, L.; Li, S.; Zhao, Z. Ultra-High-Performance Liquid Chromatography-Electrospray Ionization-High-Resolution Mass Spectrometry for Distinguishing the Origin of Ellagic Acid Extracts: Pomegranate Peels or Gallnuts. *Mol. Basel Switz.* **2024**, *29*, 666, doi:10.3390/molecules29030666.
61. Siheri, W.; Ebiloma, G.U.; Igoli, J.O.; Gray, A.I.; Biddau, M.; Akrachalanont, P.; Alenezi, S.; Alwashih, M.A.; Edrada-Ebel, R.; Muller, S.; et al. Isolation of a Novel Flavanonol and an Alkylresorcinol with Highly Potent Anti-Trypanosomal Activity from Libyan Propolis. *Mol. Basel Switz.* **2019**, *24*, 1041, doi:10.3390/molecules24061041.
62. Silva, A.P.M. da; Silva, G.S. da; Oiram Filho, F.; Silva, M.F.S.; Zocolo, G.J.; Brito, E.S. de Structural Characterization and in Vitro and in Silico Studies on the Anti-α-Glucosidase Activity of Anacardic Acids from Anacardium Occidentale. *Foods* **2024**, *13*, 4107, doi:10.3390/foods13244107.
63. Irie, J.; Murata, M.; Homma, S. Glycerol-3-Phosphate Dehydrogenase Inhibitors, Anacardic Acids, from Ginkgo Biloba. *Biosci. Biotechnol. Biochem.* **1996**, *60*, 240–243, doi:10.1271/bbb.60.240.
